# Phytochemicals in Alzheimer’s Disease Prevention and Management: Molecular Mechanisms, Therapeutic Potential, Translational Challenges, and Emerging Research Directions

**DOI:** 10.3390/ijms27146329

**Published:** 2026-07-16

**Authors:** Muhammad Sohail Khan, Imran Zafar, Jean C. Bopassa

**Affiliations:** 1 College of Korean Medicine, Gachon University, 1342 Seongnamdaero, Seongnam 13120, Republic of Korea; 2 Department of Biochemistry and Biotechnology, The University of Faisalabad (TUF), Faisalabad 38000, Punjab, Pakistan; 3 Department of Cellular and Integrative Physiology, School of Medicine, University of Texas Health Science Center at San Antonio (UTHSCSA), 7703 Floyd Curl Dr., San Antonio, TX 78229, USA

**Keywords:** Alzheimer’s disease, phytochemicals, neuroprotection, oxidative stress, neuroinflammation, amyloid-β aggregation, polyphenols, blood–brain barrier

## Abstract

Alzheimer’s disease (AD) is the most common neurodegenerative disorder and a leading cause of dementia worldwide, characterized by progressive cognitive decline, memory impairment, and neuronal loss. The pathological hallmarks of AD include extracellular accumulation of amyloid-β (Aβ) plaques, intracellular neurofibrillary tangles composed of hyperphosphorylated tau protein, chronic neuroinflammation, oxidative stress, mitochondrial dysfunction, and synaptic degeneration. Current symptomatic therapies provide modest clinical benefits, while recently approved amyloid-targeting monoclonal antibodies, such as lecanemab and donanemab, can slow decline in selected early-stage AD patients but do not cure the disease and are associated with safety, access, and cost concerns. This narrative review summarizes mechanistic evidence from in vitro and in vivo *studies* and distinguishes preclinical promise from validated clinical utility. Phytochemicals, including polyphenols, flavonoids, alkaloids, terpenoids, and carotenoids, demonstrate neuroprotective effects through antioxidant activity, anti-inflammatory modulation, inhibition of amyloid aggregation, regulation of tau phosphorylation, and support of mitochondria and synapses. Evidence from experimental models suggests that several phytochemicals may help slow AD pathology and improve cognitive function, but clinical translation remains limited due to poor bioavailability, inadequate blood–brain barrier (BBB) penetration, and a lack of large-scale clinical trials. This review highlights critical research gaps and emerging strategies to facilitate phytochemical-based preventive and therapeutic approaches in AD.

## 1. Introduction

Alzheimer’s disease (AD) accounts for 60–80% of dementia cases worldwide and is characterised by progressive cognitive and memory decline due to neuronal loss and synaptic dysfunction [1]. It is marked by the progressive deterioration of mental functions, memory, and working capacity of the brain caused by the loss of neurons and impairment of the functions of synapses in major brain areas, in particular, the hippocampus, entorhinal cortex, and association cortices [2,3]. The main pathological characteristics of AD include extracellular deposition of amyloid-β (Aβ) peptides, formation of senile plaques, and intracellular deposition of hyperphosphorylated tau protein, leading to neurofibrillary tangles. Such pathological changes impair neuronal communication and synaptic plasticity, which eventually causes massive neurodegeneration and cognitive decline [4].

Despite the central role of the amyloid cascade hypothesis, emerging evidence indicates that AD is multifactorial, involving oxidative stress, mitochondrial dysfunction, chronic neuroinflammation, and impaired proteostasis [5,6]. The accumulation of reactive oxygen species (ROS) leads to lipid peroxidation, DNA damage, and protein oxidation, thereby hastening neuronal injury. Mitochondrial dysfunction further impairs cellular energy metabolism and increases oxidative stress, exacerbating neurodegenerative mechanisms [5]. Moreover, sustained stimulation of microglia and astrocytes leads to the release of pro-inflammatory cytokines and reactive nitrogen species (RNS), which disrupt synaptic function and cause neuronal loss, and facilitate the accumulation of amyloid and the progression of tau [6]. The accumulation of toxic protein aggregates contributing to neuronal damage is also caused by impairment of protein-clearance mechanisms, including autophagy and the ubiquitin-proteasome pathway [7].

Despite decades of research, available AD treatments remain limited. Cholinesterase inhibitors and memantine provide mainly symptomatic benefit, whereas newer amyloid-targeting antibodies offer modest disease-slowing effects in selected early-stage patients but require careful safety monitoring. Current medicines, such as acetylcholinesterase inhibitors and NMDA receptor blockers, are largely symptomatic and do not prevent disease onset. Recently developed anti-amyloid monoclonal antibodies have shown a slight clinical improvement but are associated with high costs and potential safety risks [8]. As a result, the need to investigate other therapeutic approaches that could address multiple pathogenic mechanisms simultaneously is increasing.

AD is one of the critical worldwide issues of global concern, especially with the swift ageing of the population. Neurological conditions are now among the leading global causes of disability-adjusted life-years and death [9]. 57 million people were living with dementia in 2021; AD contributes approximately 60–80% of cases; projections estimate major growth by 2050 [10,11]. Notably, pathological changes associated with AD can begin decades before clinical manifestations, underscoring the importance of preventive strategies targeting early molecular mediators of neurodegeneration [12].

**Figure 1:**
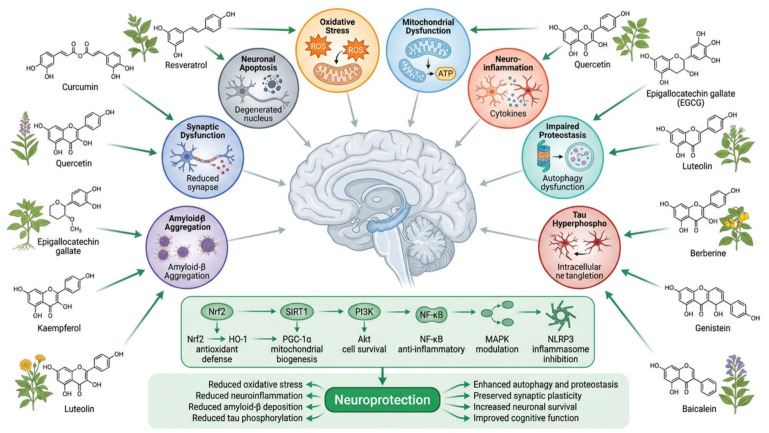
Schematic Overview of Phytochemical-Mediated Neuroprotective Mechanisms in Alzheimer’s Disease.

Over the last few years, great interest has been paid to plant-based bioactive compounds as potential preventive and therapeutic agents for AD. Phytochemicals, such as polyphenols, flavonoids, alkaloids, terpenoids, and sulfur-containing compounds, exhibit diverse biological activities in neurodegeneration [13]. Most phytochemicals have multitarget effects, whereas conventional drugs usually have a single molecular target. Antioxidants like resveratrol, curcumin, and epigallocatechin gallate (EGCG) exhibit powerful antioxidant effects by scavenging ROS and activating the nuclear factor erythroid 2-related factor 2 (Nrf2) signaling pathway, thereby boosting endogenous antioxidant defenses [13]. Moreover, various phytochemicals have anti-inflammatory properties that regulate signaling pathways, including NF-κB, MAPK, and PI3K/Akt, thereby reducing cytokine release and microglial activation [14]. Several natural products have been demonstrated to prevent amyloid-β aggregation and tau hyperphosphorylation, enhance mitochondrial activity, and promote neuronal survival [15]. The major molecular mechanisms by which phytochemicals may modulate AD pathogenesis are schematically summarized in Figure 1.

Although encouraging mechanistic results are available, the clinical translation of phytochemicals for the treatment of AD has been limited. Numerous compounds exhibit poor pharmacokinetics, low oral bioavailability, limited metabolism, and poor penetration across the blood–brain barrier (BBB). The heterogeneity in source, extraction, and formulation strategies for plants further complicates standardization and reproducibility [13]. In addition, the majority of existing evidence is derived from in vitro and animal studies, and properly designed human clinical trials are relatively few [15].

Unlike earlier reviews that primarily catalog phytochemicals, this review integrates molecular mechanisms with translational readiness, limitations in clinical evidence, formulation barriers, and emerging delivery/computational strategies. Thus, the current review aims to provide a literature review of the potential role of phytochemicals in the prevention and management of AD. This review discusses the large classes of plant-derived bioactive compounds, such as polyphenols, flavonoids, alkaloids, and terpenoids, and their potential to affect key molecular pathways in AD pathogenesis, including oxidative stress, neuroinflammation, mitochondrial dysfunction, amyloid-β aggregation, and tau hyperphosphorylation. Moreover, the review synthesizes existing experimental and clinical data on the neuroprotective effects of phytochemicals, along with observations on critical issues, knowledge gaps, and priority areas for future research to enhance their therapeutic translation.

## 2. Review Methodology

This article was prepared as a narrative review to synthesize current evidence on phytochemicals relevant to the prevention and management of AD. Literature was searched in PubMed/MEDLINE, Scopus, Web of Science, Google Scholar, and ClinicalTrials.gov using combinations of the terms “*Alzheimer’s disease*”, “*phytochemicals*”, “*polyphenols*”, “*flavonoids*”, “*curcumin*”, “*resveratrol*”, “*quercetin*”, “*EGCG*”, “*berberine*”, “*ginsenosides*”, “*sulforaphane*”, “*oxidative stress*”, “*neuroinflammation*”, “*amyloid-beta*”, “*tau phosphorylation*”, “*blood-brain barrier*”, “*nanodelivery*”, *and* “*clinical trial*”. Priority was given to peer-reviewed original studies, randomized or controlled clinical studies where available, systematic reviews, and recent mechanistic reviews published in English. Studies were included when they addressed AD-related molecular mechanisms, preclinical neuroprotective effects, pharmacokinetic limitations, formulation strategies, or clinical translation of phytochemical-based interventions. Studies not directly related to AD, phytochemicals, neurodegeneration, or translational development were excluded.

### Barriers to Clinical Translation

A recurring paradox in this field is that many phytochemicals are highly effective in cellular and animal models yet fail to reproduce these benefits in humans, and the reasons for this translational gap deserve explicit discussion rather than being left implicit. Several factors converge. First, pharmacokinetic attrition is severe: compounds such as curcumin, resveratrol, and EGCG undergo extensive first-pass glucuronidation and sulfation, are rapidly metabolised, and reach only nanomolar plasma and brain concentrations, frequently far below the micromolar levels used in vitro, so that the exposures that drive efficacy in the dish are simply never achieved at the target tissue. Second, limited and variable blood–brain barrier (BBB) penetration means that even systemically available compounds may not reach therapeutic concentrations in the brain parenchyma. Third, preclinical models are themselves imperfect predictors: transgenic rodents typically overexpress single familial mutations, develop pathology on a compressed timescale, and lack the multimorbidity, ageing, and genetic heterogeneity of sporadic human AD, so a compound optimised against a mouse phenotype may address a target that is of limited relevance to patients. Fourth, most positive preclinical studies administer compounds early and prophylactically, whereas human trials necessarily recruit patients with established, often advanced pathology, by which point the targeted upstream processes may no longer be modifiable. Finally, methodological factors, non-standardized extracts, heterogeneous dosing and formulations, small and short trials, and inconsistent biomarker or cognitive endpoints reduce statistical power and reproducibility. Recognizing these barriers is essential for interpreting the evidence presented below and frames the formulation, delivery, and trial-design strategies as discussed in this review.

## 3. Pathophysiological Basis of Alzheimer’s Disease

**Figure 2:**
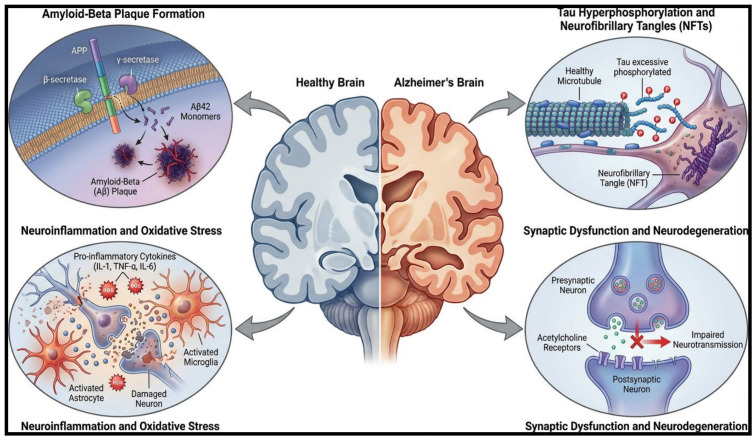
Pathogenic mechanisms and phytochemical targets in AD. The schematic illustrates the interconnected pathways driving AD progression, including amyloid-β (Aβ) aggregation, tau hyperphosphorylation, oxidative stress, mitochondrial dysfunction, neuroinflammation, proteostatic failure, and synaptic loss. These self-amplifying processes converge on neurodegeneration and cognitive decline, highlighting key multitarget sites for phytochemical intervention. *Figure Note*: Aβ, amyloid-β; APP, amyloid precursor protein; ROS, reactive oxygen species; RNS, reactive nitrogen species; NFTs, neurofibrillary tangles. (Internal figure labels—including “mitochondrial” and “aggregation”—have been corrected in the regenerated high-resolution figure file).

AD is a multifaceted disease in which several molecular and cellular defects work together to promote a progressive neurodegenerative process and cognitive impairment. The primary pathological mechanisms are extracellular accumulation of amyloid-β (Aβ) plaques, intracellular neurofibrillary tangles containing hyperphosphorylated tau protein, oxidative stress, mitochondrial dysfunction, neuroinflammation, disrupted proteostasis, and synaptic degeneration [16,17]. Aβ aggregation interferes with neuronal communication, promotes oxidative stress, and activates inflammatory signalling pathways, while tau hyperphosphorylation destabilises the microtubules and hampers axonal transport, thus resulting in neuronal dysfunction and cell death. Excessive oxidative stress due to ROS-induced damage to cellular lipids, proteins, and nucleic acids, along with mitochondrial dysfunction, further increases neuronal injury by decreasing ATP production, regulating calcium levels, and disrupting apoptotic mechanisms. Activated microglia and astrocytes produce pro-inflammatory cytokines and neurotoxic mediators that further exacerbate neuronal damage and impair the clearance of toxic protein aggregates. Problems with protein folding, such as impaired ubiquitin-proteasome and autophagy–lysosomal function, can lead to misfolded proteins and greater neuronal vulnerability. The pathological processes are interdependent and feed into each other in a cycle that increasingly compromises the integrity and function of synapses and neural networks, leading to memory loss and cognitive decline. The same kind of overlapping molecular mechanisms is seen in other neurodegenerative diseases such as Parkinson’s disease, Huntington’s disease, and amyotrophic lateral sclerosis. The main pathogenic mechanisms leading to neuronal loss in AD are shown in Figure 2, and the major molecular and cellular mechanisms in neurodegenerative pathogenesis are summarised in Table 1.

### 3.1. Amyloid-β Pathology and Tau Hyperphosphorylation

Abnormal production, impaired clearance, and aggregation of amyloid-β (Aβ) peptides generated from amyloid precursor protein (APP) are among the best-characterised events in AD pathogenesis. Under physiological conditions, APP cleavage is primarily mediated by α-secretase in the non-amyloidogenic pathway, yielding soluble peptides that do not form toxic aggregates. In AD pathology, however, the sequential cleavage of APP by β-secretase (BACE1) and γ-secretase to generate amyloid-β peptides occurs preferentially, with a more aggregation-prone isoform, Aβ42, being the major one [31]. An imbalance between amyloid production and clearance leads to the accumulation of amyloid-β oligomers, protofibrils, and plaques in the brain. Soluble amyloid-β oligomers are highly neurotoxic, disrupting neuronal membranes, impairing synaptic signalling, and inhibiting long-term potentiation (LTP). They also disturb calcium balance and increase oxidative stress. In addition, amyloid-β aggregates activate microglia and astrocytes, causing inflammation that further damages neurons [16].

Hyperphosphorylated tau protein accumulation is closely associated with amyloid pathology. Tau is a microtubule-associated protein that stabilizes the neuronal cytoskeleton and supports axonal transport. Excessive tau phosphorylation in AD results from dysregulation of kinases such as glycogen synthase kinase-3β (GSK-3β) and cyclin-dependent kinase-5 (CDK5). This pathological phosphorylation causes tau to dissociate from microtubules and aggregate into paired helical filaments, forming neurofibrillary tangles [32]. The growth of neurofibrillary tangles disrupts neurotransmitter systems, impairs synaptic connections, and eventually leads to neuronal death. Notably, tau pathology distribution and density are more strongly linked to cognitive impairment than amyloid plaque burden, suggesting that tau-mediated neurodegeneration is a key contributor to disease development. There exists growing evidence that amyloid pathology could serve as an upstream stimulus that increases the rate of tau hyperphosphorylation and aggregation, thus connecting the two pathological phenomena in a shared neurodegenerative cascade.

### 3.2. Oxidative Stress and Mitochondrial Dysfunction

Oxidative stress is another central mechanism contributing to neuronal injury in AD. The human brain consumes nearly a fifth of the body’s total oxygen, even though it accounts for approximately 2% of the body’s mass, making neuronal tissue especially vulnerable to oxidative damage. Overproduction of ROS and RNS leads to lipid peroxidation, protein oxidation, and DNA damage, all of which impair neuronal integrity and cellular signalling pathways [33]. Mitochondrial dysfunction is central to the development of oxidative stress in AD. ATP is produced by oxidative phosphorylation in mitochondria. However, in AD, the mitochondrial respiratory chain becomes dysfunctional, leading to defects in complexes I and IV. This limitation reduces cellular energy production while increasing ROS production. It has also been revealed that amyloid-β peptides may localize to mitochondria, where they interact with mitochondrial membranes and enzymes, further impairing energy metabolism and exacerbating oxidative damage [33]. Moreover, mitochondrial abnormalities destabilize calcium homeostasis and initiate intrinsic apoptotic pathways, leading to cytochrome c release and caspase activation. These events eventually lead to neuronal cell death and brain atrophy. Oxidative damage also affects lipid membranes and synaptic proteins, thereby increasing synaptic connectivity loss, as observed in AD. Notably, oxidative stress can increase amyloid-β production by elevating BACE1 expression and facilitating tau phosphorylation, linking metabolic dysfunction to protein aggregation pathways.

### 3.3. Neuroinflammation, Impaired Proteostasis, and Synaptic Degeneration

Chronic neuroinflammation is increasingly recognized as a central contributor to AD pathogenesis. In response to amyloid-β deposits and neuronal damage, microglia and astrocytes are activated and secrete pro-inflammatory cytokines, including tumor necrosis factor-α (TNF-α), interleukin-1β (IL-1β), and interleukin-6 (IL-6). Although the initial triggering of glial cells can help clear amyloid deposits, prolonged activation triggers chronic inflammatory signals that destroy neurons and worsen disease progression [34]. The nuclear factor-κB (NF-κB) and NLRP3 inflammasome pathways are highly involved in the inflammatory response. Stimulation of these pathways leads to the release of inflammatory cytokines and ROS, exacerbating oxidative stress and neuronal dysfunction. Genetic research has also identified several immune-related genes, such as TREM2 and CD33, that mediate microglial responses to amyloid buildup, underscoring the role of immune dysregulation in AD pathogenesis. Several phytochemicals may also exhibit anti-inflammatory activity by suppressing the NF-κB signaling pathway and preventing the synthesis of pro-inflammatory molecules, including inducible nitric oxide synthase (iNOS) and cyclooxygenase-2. Microglial stimulation and the generation of inflammatory cytokines in experimental models of AD have been significantly reduced by curcumin, resveratrol, quercetin, and luteolin [35,36,37].

Another significant factor in AD development is the loss of cellular proteostasis systems that clear misfolded proteins. The ubiquitin-proteasome and the autophagy–lysosomal pathways are the two common pathways that maintain protein quality in neurons. Now, however, in AD, these systems progressively become dysfunctional, and toxic protein aggregates such as amyloid-β and hyperphosphorylated tau accumulate in neuronal cells [38]. Impaired autophagic lysosomes and vacuoles in neurons result from defective autophagic degradation, further exacerbating cellular stress and neurodegeneration. Accumulated results of amyloid toxicity, tau pathology, oxidative stress, mitochondrial dysfunction, neuroinflammation, and weakened proteostasis finally lead to synaptic degeneration. Synapse loss is thought to be one of the first pathological processes in AD and one that is highly correlated with cognitive loss. Soluble amyloid-β oligomers interfere with the neurotransmission of glutamatergic neurons, inhibit synaptic plasticity, and cause excitotoxicity by overactivating NMDA receptors [31]. Gradual synapse degeneration ultimately leads to neuronal apoptosis and the degeneration of extensive cortical and hippocampal networks involved in memory and cognition. The multi-domain, interrelated pathogenic processes are used to demonstrate the complexity of AD and to emphasize the shortcomings of therapeutic approaches based on a single molecular pathway. AD is multifactorial and thus has led to the development of multitarget medications that can modulate oxidative stress, inflammation, mitochondrial dysfunction, and protein aggregation, all of which can be altered simultaneously.

## 4. Major Classes of Neuroprotective Phytochemicals

Phytochemicals with potential relevance to AD can be broadly classified into polyphenols, flavonoids, alkaloids, terpenoids, carotenoids, and other plant-derived bioactive compounds, each of which includes representative molecules with distinct chemical characteristics but partially overlapping neuroprotective functions. As summarised in Table 2, this classification is useful not only from a phytochemical or pharmacognostic perspective but also for organising the therapeutic landscape of candidate compounds by their dominant molecular targets, translational maturity, and experimental support. Although these compounds differ structurally, many converge functionally on the same pathological processes that drive AD, particularly oxidative stress, neuroinflammation, mitochondrial dysfunction, amyloid-β accumulation, tau dysregulation, impaired proteostasis, and synaptic injury.

Among the major phytochemical classes investigated in AD, polyphenols are the most extensively studied. This predominance is supported by bibliometric analyses of the AD literature, which demonstrate that flavonoids constitute the most frequently reported polyphenolic subclass, while curcumin, resveratrol, epigallocatechin-3-gallate (EGCG), and quercetin are the most extensively investigated individual phytochemicals in the field [39]. Furthermore, recent evidence syntheses and knowledge-mapping studies continue to identify polyphenols as a dominant focus of neurodegeneration research owing to their multitarget pharmacological properties, including antioxidant, anti-inflammatory, anti-amyloidogenic, and neuroprotective activities [40,41].

This class encompasses several extensively characterized compounds, including resveratrol, curcumin, and EGCG, each of which exhibits pleiotropic mechanisms relevant to AD pathogenesis. Resveratrol has been implicated in modulating SIRT1/AMPK-associated signaling pathways, mitochondrial function, autophagy, oxidative stress responses, and neuroinflammatory processes. Curcumin has consistently demonstrated the capacity to interfere with amyloid-β aggregation, attenuate tau hyperphosphorylation, regulate redox homeostasis, and suppress NF-κB-mediated inflammatory signaling [42,43]. EGCG is a prominent catechin in green tea, and its anti-aggregatory effects and ability to modulate oxidative and inflammatory signaling are particularly powerful, but its effectiveness in the translational setting is hampered by stability and pharmacokinetic limitations [44]. Taken together, these compounds have become core reference molecules in the field because each exemplifies the role of a single phytochemical class across multiple mechanistic levels of AD pathology.

**Table 2:** Classification of phytochemicals with neuroprotective potential in neurodegenerative disorders: chemical classes, principal mechanisms of action, representative original evidence, and translational relevance.

Part	Phytochemical Class/Exemplar	Chemical Subclass and Common Plant Source(s)	Dominant Neuroprotective Mechanisms Relevant to NDDs	Representative Original Evidence	Translational Relevance/Main Limitation	Citations
1	Polyphenols—Resveratrol	Stilbene polyphenol; grapes, berries, peanuts	Activates SIRT1/AMPK, suppresses NF-κB-linked inflammation, reduces oxidative stress, modulates Aβ/tau biology, supports mitochondrial function and autophagy	In a 52-week phase 2 randomized placebo-controlled trial in mild-to-moderate AD, resveratrol showed acceptable safety and biomarker effects, but not definitive clinical efficacy	Strong mechanistic rationale; human data exist, but exposure and dose requirements remain limiting	[45]
1	Polyphenols—Curcumin	Diarylheptanoid polyphenol; *Curcuma longa* (turmeric)	Anti-amyloidogenic, anti-tau, Nrf2/HO-1 activation, NF-κB suppression, anti-inflammatory, anti-apoptotic, antioxidant	A 24-week randomized double-blind placebo-controlled AD study found curcumin was tolerated, but clinical/biomarker efficacy was limited in that formulation	One of the most studied compounds experimentally; a major barrier is poor bioavailability and formulation dependence	[46]
1	Polyphenols—EGCG	Catechin-type polyphenol; green tea (*Camellia sinensis*)	Inhibits Aβ and α-syn aggregation, scavenges ROS, chelates redox-active metals, modulates MAPK/Nrf2 and neuroinflammatory signaling	Experimental work showed EGCG inhibits α-synuclein aggregation and remodels toxic species relevant to synucleinopathies	Strong anti-aggregation profile; clinical translation remains constrained by stability, metabolism, and target exposure	[47]
2	Flavonoids—Quercetin	Flavonol; onions, apples, berries, leafy vegetables	Antioxidant, NLRP3/NF-κB suppression, anti-apoptotic, mitochondrial protection, modulation of amyloidogenesis and synaptic injury	In aged 3xTg-AD mice, quercetin improved cognitive/emotional outcomes and reduced AD-like pathology	Broad experimental efficacy; limited by low oral bioavailability and rapid metabolism	[48]
2	Flavonoids—Catechin	Flavan-3-ol; tea, cocoa, grapes, apples	Antioxidant, anti-inflammatory, preservation of synaptic plasticity, modulation of MAPK/PI3K-Akt and mitochondrial redox signaling	Evidence is mainly preclinical, with green-tea catechin studies showing protection against oxidative injury, inflammatory activation, and proteotoxic stress in neuronal models	Mechanistically attractive but often discussed together with EGCG rather than as a stand-alone clinical candidate	[49]
2	Flavonoids—Kaempferol	Flavonol; tea, kale, broccoli, beans, berries	Anti-oxidative, anti-inflammatory, anti-apoptotic, anti-amyloid/anti-tau/anti-α-syn tendencies, mitochondrial stabilization, cholinesterase-related effects	Recent synthesis of in vitro/in vivo evidence indicates benefit across AD, PD, HD, and ALS models, with especially recurrent effects on aggregation, microglia, and mitochondrial integrity	Mainly preclinical; pharmacokinetic optimization is still needed	[50]
3	Alkaloids—Galantamine	Amaryllidaceae alkaloid; Galanthus, Leucojum, Narcissus spp.	Reversible acetylcholinesterase inhibition plus positive allosteric modulation of nicotinic acetylcholine receptors; symptomatic cognitive support	In a 2-year randomized placebo-controlled AD study, galantamine showed sustained symptomatic efficacy/survival-related evaluation in mild-to-moderately severe AD	Highest clinical maturity in this table; already approved, but mainly symptomatic rather than disease-modifying	[51,52]
3	Alkaloids—Berberine	Protoberberine isoquinoline alkaloid; Berberis, Coptis, Hydrastis spp.	Anti-inflammatory, anti-amyloidogenic, pro-autophagic, anti-tau, anti-oxidative, mitochondrial support, gut–brain/metabolic modulation	In 3xTg-AD models, berberine improved cognition, promoted autophagic clearance, and reduced Aβ or tau-related pathology	Strong preclinical profile; key limitations are bioavailability, dose optimization, and lack of robust late-phase clinical data	[53,54,55]
4	Terpenoids—Ginsenosides	Triterpenoid saponins: Panax ginseng, P. notoginseng, P. quinquefolius	Anti-inflammatory, antioxidant, anti-apoptotic, anti-excitotoxic, mitochondrial protection, autophagy/mitophagy regulation, anti-amyloid effects	Ginsenoside Rg1 improved pathology in AD-model mice; ginsenoside Rd and other congeners are also extensively studied in CNS injury and neurodegeneration	Large preclinical literature; compound-specific pharmacology varies widely across Rg1, Rb1, Rd, Rg2, CK, etc.	[56,57,58]
4	Terpenoids—Carotenoids	Tetraterpenoids: carotene/xanthophyll family from tomato, carrot, saffron, microalgae, marigold, seafood; examples include lycopene, lutein, astaxanthin	ROS quenching, membrane stabilization, anti-inflammatory signaling, anti-amyloid effects, mitochondrial protection	Astaxanthin improved cognitive deficits in APP/PS1 mice; the carotenoid literature also includes mechanistic work on Aβ interaction and antioxidant neuroprotection	Class is chemically diverse; BBB exposure and formulation vary markedly by carotenoid	[59,60,61]
5	Other plant bioactives—Sulforaphane	Isothiocyanate derived from glucoraphanin; broccoli and other crucifers	Potent Nrf2/ARE inducer, anti-inflammatory, anti-apoptotic, mitochondrial protection, support of proteostasis, and detoxification enzymes	In a 6-OHDA mouse PD model, sulforaphane improved motor deficits and reduced dopaminergic neurotoxicity	Strong redox biology and reproducible preclinical signal; limitations relate to formulation, dosing, and human CNS outcome data	[62]
5	Other plant bioactives—Luteolin	Chemically, a flavone (often discussed separately because of strong anti-neuroinflammatory activity); celery, parsley, chamomile, thyme, peppers	Anti-neuroinflammatory, ER-stress attenuation, anti-oxidative, anti-amyloidogenic, synaptic protection, microglial modulation	In 3xTg-AD mice, luteolin alleviated cognitive impairment and reduced ER stress-dependent neuroinflammation	Strong mechanistic profile, but clinical confirmation remains limited	[35,63]
5	Related compounds often added in this section	Anthocyanins, baicalein, apigenin, xanthohumol, β-caryophyllene, plant polysaccharides, tannins, phenolic acids	Commonly converge on ROS suppression, inflammasome control, autophagy, synaptic protection, and proteostasis	Evidence is predominantly preclinical and compound-specific	Useful to mention in a review, but best handled in a separate “emerging phytochemicals” table if you want stricter mechanistic granularity	[64,65]

The translational profile of representative neuroprotective phytochemicals demonstrates considerable heterogeneity in their readiness for AD therapy, particularly with respect to blood–brain barrier (BBB) penetration, oral bioavailability, clinical validation, and overall evidence strength. As summarized in Table 3, galantamine represents the most advanced compound because it has already achieved routine clinical use as an approved symptomatic treatment for AD, whereas phytochemicals such as resveratrol, huperzine A, curcumin, genistein, saffron-derived compounds, and macular carotenoids have progressed into human clinical evaluation but still show variable or non-definitive therapeutic outcomes. In contrast, compounds including EGCG, quercetin, kaempferol, berberine, ginsenoside Rg1, sulforaphane, luteolin, apigenin, naringenin, fisetin, baicalein, ferulic acid, astaxanthin, oleocanthal, with anoside IV, bacoside A, and urolithin A remain largely supported by preclinical evidence, despite showing promising anti-amyloid, anti-tau, antioxidant, anti-inflammatory, mitochondrial, and neuroprotective effects. Earlier studies highlight that poor oral bioavailability, uncertain human brain exposure, limited BBB pharmacokinetic validation, and insufficient large-scale randomized clinical trials remain major barriers to the successful translation of phytochemical-based interventions for AD and other neurodegenerative disorders.

Another key group with significant neuroprotective potential is flavonoids. Preclinical results have demonstrated the usefulness of representative compounds, including quercetin, catechin, kaempferol, and luteolin, which are antioxidants, anti-inflammatory, anti-apoptotic, and synaptic-supportive. AD-like pathology reduction and improvements in cognitive and emotional outcomes in transgenic mouse models have been reported with quercetin, whereas luteolin has become of particular interest due to its strong anti-neuroinflammatory properties and its capacity to reduce inflammatory signaling via endoplasmic reticulum stress [35,48]. Even when flavonoids are discussed collectively because of their structural similarity, their biological effects are not equal, and some appear to exert more potent effects on glial stimulation, synaptic plasticity, or mitochondrial stability than others.

Galantamine is a plant-derived alkaloid and an approved symptomatic therapy; however, its clinical role is cholinergic symptom management rather than modification of amyloid/tau-driven disease progression [85]. Berberine is another alkaloid that has demonstrated preclinical benefits for reducing amyloid burden, tau pathology, autophagic clearance, neuroinflammation, and cerebral perfusion [54,55]. The significance of this difference lies in the fact that phytochemicals can occupy a variety of translational positions, ranging from symptomatic drugs in use but in need of further development to experimental multitarget compounds undergoing mechanistic testing.

Other phytochemicals that play a significant role in the phytochemical landscape of AD include terpenoids and carotenoid-related compounds. The Panax-derived major triterpenoid saponins, ginsenosides, have been shown to exert anti-inflammatory, anti-apoptotic, anti-excitotoxic, and mitochondrially supportive effects in experimental neurodegenerative models, with ginsenoside Rg1 demonstrating a beneficial effect on Alzheimer-like pathology in animals [56,57]. The relevance of carotenoids (in particular, astaxanthin and lutein) lies in their membrane-stabilising and ROS-quenching effects, and some research indicates that they can also mitigate amyloid-associated and inflammatory damage in the AD brain [59].

Another type is other plant-derived bioactives, which are not necessarily easy to classify in the above major categories but are of growing mechanistic interest. Sulforaphane, in particular, is an isothiocyanate that is produced by cruciferous vegetables and is extensively researched as a potent activator of the Nrf2/ARE pathway, with further actions on inflammation, proteostasis, detoxification enzymes, and mitochondrial resilience [37,60,62]. Likewise, apigenin, baicalein, anthocyanins, xanthohumol, phenolic acids, and certain plant polysaccharides are among the compounds that have begun to find their place on the list of neuroprotective compounds under discussion, even though the quality of evidence supporting them varies significantly [64].

## 5. Molecular Mechanisms of Phytochemical Neuroprotection

Phytochemicals have attracted significant interest as potential preventive and therapeutic agents against AD due to their capacity to regulate various disease-related pathological pathways that drive disease onset and progression. In contrast to pharmacological compounds, which are usually specific to a given molecular mechanism, most plant-based bioactive compounds exhibit pleiotropic effects across multiple interconnected pathways involved in neurodegeneration. AD is a multidimensional pathological process characterized by the accumulation of amyloid-β, hyperphosphorylation of tau, oxidative stress, mitochondrial dysfunction, chronic neuroinflammation, impaired proteolysis, synaptic loss, and neuronal necrosis [16,17,19]. These mechanisms have been found to work together and amplify each other, making approaches to therapy that focus on a single pathway less successful. To fit into this paradigm, multitarget neuroprotective phytochemicals are emerging as a focus in AD prevention and management [65,86].

A great number of phytochemicals have been revealed to have neuroprotective properties in animal models of AD. These are polyphenols, flavonoids, alkaloids, terpenoids, carotenoids, and isothiocyanates. The most studied of the compounds include curcumin, resveratrol, EGCG, quercetin, berberine, luteolin, ginsenosides, and sulforaphane. Even though they are structurally different, these molecules overlap in several biological processes that are considered to be relevant in neurodegeneration, in particular, controlling oxidative stress, inhibiting neuroinflammation, regulating amyloid and tau pathophysiology, maintaining mitochondrial functions, promoting proteolysis, and sustaining neuronal signaling [87].

**Figure 3:**
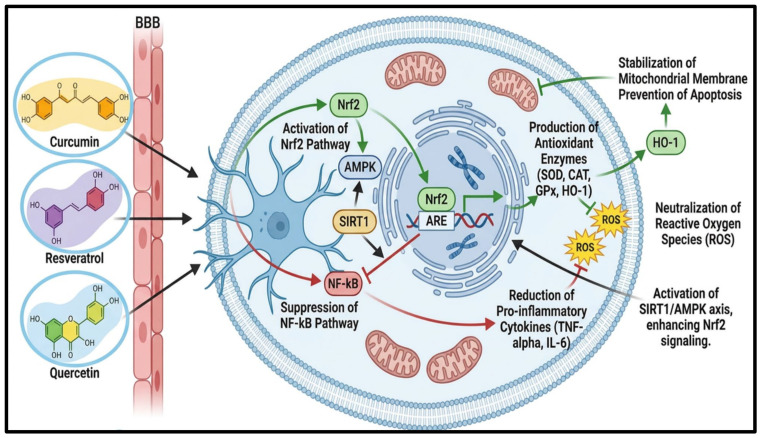
Molecular Signaling Pathways Underlying Phytochemical-Mediated Neuroprotection in Alzheimer’s Disease.

On the molecular level, various phytochemicals have an effect on intracellular signaling pathways, such as nuclear factor erythroid-2-related factor 2 (Nrf2), nuclear factor-κB (NF-κB), AMP-activated protein kinase (AMPK), sirtuin-1 (SIRT1), phosphoinositide 3-kinase/protein kinase B (PI3K/Akt), and brain-derived neurotrophic factor (BDNF) signaling pathways. Phytochemicals such as curcumin, resveratrol, and quercetin, as depicted in Figure 3, have the potential to activate the Nrf2 signaling pathway and, consequently, increase the expression of antioxidant enzymes, such as superoxide dismutase (SOD), catalase (CAT), and glutathione peroxidase (GPx), thereby neutralizing ROS. The coloured rings highlighted on the chemical structures denote the phytochemical subclass (polyphenol, flavonoid, alkaloid, terpenoid, isothiocyanate) rather than any specific reactive moiety. At the same time, these compounds can inhibit NF-κB signalling, which drives inflammatory responses, thereby reducing the expression of pro-inflammatory cytokines, including tumour necrosis factor-alpha (TNF-α) and interleukin-6 (IL-6). The coordinated mechanisms of phytochemicals stabilize mitochondrial function, inhibit apoptosis, and enhance neuronal survival and synaptic integrity [88,89].

### 5.1. Regulation of Nrf2-Mediated Oxidative Stress and Mitochondrial Dysfunction

The human brain accounts for approximately 2% of body mass but consumes nearly a fifth of total oxygen, making neurons particularly vulnerable to oxidative stress. The overproduction of ROS and RNS leads to lipid peroxidation, protein oxidation, DNA damage, and mitochondrial dysfunction, ultimately resulting in neuronal death and synaptic dysfunction [5,33].

Phytochemicals may counter oxidative stress through both direct radical-scavenging activity and indirect activation of endogenous antioxidant and anti-inflammatory signaling pathways. In AD-relevant experimental systems, curcumin has been reported to enhance HO-1/Nrf2-associated cytoprotection in APPswe-transfected SH-SY5Y cells, while quercetin activates SIRT1/Nrf2/HO-1 signaling in Aβ25–35-induced PC12 cells and promotes NRF2/HO1-mediated microglial polarization in Aβ1–42-based AD models [90,91,92]. Sulforaphane is also closely linked to NRF2/ARE activation in AD models, where NRF2 activation reduces BACE1/BACE1-AS expression, decreases Aβ production, and improves cognitive deficits [93]. Resveratrol supports mitochondrial and antioxidant resilience mainly through SIRT1-related signaling; in an AD/tauopathy model, resveratrol reduced hippocampal neurodegeneration and decreased acetylation of SIRT1 substrates, including PGC-1α and p53 [94]. In parallel, luteolin and quercetin suppress neuroinflammatory signaling by inhibiting NF-κB and NLRP3 inflammasome-related pathways, thereby reducing Aβ-associated inflammatory injury, microglial activation, and oxidative neurotoxicity [95,96,97].

Activation of Nrf2 promotes the transcription of cytoprotective genes encoding antioxidant enzymes, including SOD, catalase, glutathione peroxidase, NAD(P)H quinone oxidoreductase-1, and heme oxygenase-1 (HO-1), which collectively strengthen cellular defense systems against oxidative stress [98,99]. Oxidative stress is closely related to mitochondrial dysfunction, which is a decisive phenomenon in neurodegeneration. In AD, dysregulation of mitochondrial respiratory chain complexes leads to reduced ATP production and increased ROS production, in a vicious cycle of oxidative damage and energy deprivation [33]. A number of phytochemicals are known to enhance mitochondrial activity by stimulating signaling pathways involved in mitochondrial biogenesis and mitochondrial metabolism. For example, resveratrol activates the SIRT1-PGC-1α pathway, thereby promoting mitochondrial biogenesis and enhancing cellular energy metabolism [100]. Likewise, mitochondrial integrity and functioning have been shown to be protected by curcumin and ginsenosides in models of neurodegenerative diseases [101,102].

Although Nrf2 activation and NF-κB inhibition are often considered beneficial in AD for their antioxidant and anti-inflammatory effects, chronic or excessive modulation of these pathways may have context-dependent adverse consequences. Persistent Nrf2 activation has been associated with altered redox homeostasis, enhanced survival of damaged or malignant cells, resistance to apoptosis, metabolic reprogramming, and chemoresistance, indicating that long-term pharmacological induction of Nrf2 should not be assumed to be uniformly protective [103,104,105,106]. Similarly, broad or sustained suppression of NF-κB signaling may be problematic because NF-κB also contributes to glial homeostasis, astrocyte–microglia communication, and Aβ clearance responses; therefore, excessive inhibition may compromise reparative neuroimmune functions in the aged brain [107,108]. In addition, several phytochemicals can modulate cytochrome P450 enzymes and drug transporters, especially CYP3A4 and P-glycoprotein, thereby increasing the risk of herb–drug interactions, altered drug exposure, toxicity, or therapeutic failure in elderly AD patients receiving polypharmacy [109,110,111,112]. Thus, the therapeutic window, dose, duration, and patient-specific medication profile should be carefully defined when proposing chronic phytochemical-based modulation of pathways in AD.

### 5.2. Mitochondrial Protection and Energy Metabolism

In neurons, mitochondria play a crucial role in cellular respiration, ATP production, calcium regulation, and modulation of apoptotic signaling [113]. Neurons are uniquely dependent on mitochondria and their function, and even slight abnormalities can have a profound effect on synaptic transmission and neuronal survival [114]. Mitochondrial dysfunction has been linked to neurodegenerative diseases, including AD, in which it manifests as deficits in oxidative phosphorylation, electron transport chain dysfunction, altered mitochondrial dynamics, and decreased bioenergetic efficiency [5]. Abnormal levels of amyloid-β and tau proteins further impair mitochondrial transport and membrane permeability, leading to neuronal energy depletion and synaptic failure. Also, mitochondrial DNA mutations and calcium imbalances are thought to lead to neuronal death via apoptotic cascades and to disrupt intracellular signaling pathways.

Multiple phytochemicals have been shown to protect mitochondrial homeostasis and neuronal energy metabolism [115]. A large catechin found in green tea is EGCG, which stabilizes the mitochondrial membrane and helps lower mitochondrial DNA oxidation [116]. Berberine stimulates AMP-activated protein kinase (AMPK), the enzyme that controls glucose uptake, fatty acid oxidation, and mitochondrial energy balance [117]. Rutin and kaempferol have also been shown to enhance mitochondrial respiration and maintain electron-transport-chain enzyme activity in neurodegenerative models [118]. In addition, phytochemicals can modulate mitochondrial quality-control mechanisms, which could help remove damaged mitochondria and prevent their accumulation [119]. Increased expression of mitochondrial fusion proteins, such as mitofusin-1 and optic atrophy-1, is also associated with improved neuronal survival and the preservation of synaptic integrity. These results indicate that phytochemicals could help maintain neuronal bioenergetics and may delay neurodegeneration by modulating mitochondrial function and energy metabolism.

The comparative evidence summarized in Table 4 indicates that phytochemicals differ substantially in the strength and specificity of their mitochondrial-protective effects in AD models. Compounds such as curcumin, quercetin, luteolin, berberine, ginsenoside Rg1, urolithin A, honokiol, salidroside, crocin, icariin, genistein, paeoniflorin, puerarin, and ferulic acid show comparatively direct evidence of mitochondrial protection in AD or AD-like experimental systems, including modulation of mitochondrial membrane potential, ATP production, oxidative phosphorylation, mitophagy, mitochondrial dynamics, cytochrome c release, and apoptosis-related pathways. In contrast, compounds such as resveratrol, sulforaphane, apigenin, naringenin, baicalein, fisetin, kaempferol, withanoside IV/sominone, and bacoside A provide supportive but more indirect evidence, because their cited mechanisms are either non-AD-specific, primarily anti-amyloid/anti-inflammatory, or not directly focused on mitochondrial endpoints. Evidence explored from earlier studies helps distinguish phytochemicals with direct mitochondrial evidence from those with broader neuroprotective or supportive mechanistic relevance, thereby reducing overinterpretation of preclinical findings.

### 5.3. Anti-Inflammatory and Glial-Modulating Effects

Neuroinflammation is now recognized as a key driver of neurodegenerative disease progression [139]. Chronic activation of microglia and astrocytes leads to a chronic inflammatory response that causes neuronal injury, synaptic impairment, and cognitive decline [140]. In pathological conditions, activated glial cells secrete excessive amounts of inflammatory cytokines, chemokines, NO, COX-2, and iNOS, all of which contribute to neuronal damage and resulting oxidative stress [141]. Chronic neuroinflammation also damages the blood–brain barrier and causes a build-up of toxic protein aggregates (such as amyloid-β plaques). In AD, activated microglia cannot effectively remove amyloid residues, thereby sustain the inflammatory signal and cause progressive neuronal injury.

Phytochemicals have strong anti-inflammatory effects, regulate glia activity, and inhibit neurotoxic inflammatory pathways [142]. Apigenin and luteolin have been found to block microglial overactivation and decrease the levels of pro-inflammatory mediators such as TNF-α, IL-1β, and IL-6 [143]. Genistein inhibits the MAPK and JAK/STAT pathways and reduces neuronal inflammation and apoptosis, thereby preventing inflammatory signaling [144]. Similarly, baicalein inhibits astrocyte activation and the release of inflammatory mediators in experimental models of neurodegenerative diseases. Other phytochemicals also induce microglial polarization toward the M2 anti-inflammatory phenotype, facilitating tissue repair, phagocytosis of toxic aggregates, and neuronal regeneration. Moreover, omega-3-based phytocompounds and polyphenols could enhance the release of neurotrophic factors such as brain-derived neurotrophic factor (BDNF), thereby facilitating neuronal recovery and synaptic plasticity [145]. These phytochemicals could have substantial therapeutic relevance for modulating glial cell activity, suppressing chronic inflammation, slowing the progression of neurodegenerative diseases, and maintaining cognitive function.

### 5.4. Modulation of Amyloid-β and Tau Pathology

The formation of amyloid-β peptides and hyperphosphorylated tau protein is the most significant pathological characteristic of AD. The abnormal cleavage of APP by β-secretase (BACE1) and γ-secretase results in amyloid-β peptides, especially the aggregation-prone Aβ42 isoform, and forms soluble oligomers and extracellular plaques. These oligomers interfere with synaptic signaling, disrupt calcium homeostasis, induce oxidative stress, and trigger inflammatory responses, thereby contributing to neuronal degeneration [24,31]. A number of phytochemicals have been found to affect amyloid pathology in various ways. Curcumin can bind amyloid-β peptides, prevent fibril formation, and stimulate plasma clearance by increasing phagocytosis and autophagy [146]. EGCG has also been found to remodel amyloid fibrils into less toxic structures and to decrease oligomer-associated toxicity [147]. Similarly, resveratrol and quercetin also decrease amyloid deposits by altering proteostasis, autophagy, and inflammatory signaling [48]. Another important neuroprotective effect of phytochemicals is the modulation of tau pathology. Hyperphosphorylation of tau destabilizes microtubules, leading to neurofibrillary tangles, which are positively associated with cognitive impairment in AD [32]. Some phytochemicals may prevent tau phosphorylation by inhibiting kinases that phosphorylate tau, including glycogen synthase kinase-3β (GSK-3β), and decrease tau aggregation. Curcumin and resveratrol have been shown to exhibit encouraging properties in the process of tau phosphorylation and tangle formation in an experimental AD model [148].

### 5.5. Proteostasis, Autophagy, and Synaptic Protection

Proteostasis is vital to neuronal survival. The Ubiquitin-proteasome system and the autophagy–lysosomal pathway are involved in the elimination of misfolded or damaged proteins in healthy neurons. But in AD, these protein quality-control mechanisms fail, leading to the formation of harmful aggregates such as amyloid-β and hyperphosphorylated tau [38]. A number of phytochemicals have been shown to increase autophagic clearance and rescue proteostasis. Berberine, resveratrol, curcumin, and sulforaphane are compounds shown to promote autophagy and lysosomal activity, remove harmful protein aggregates, and enhance neuronal survival [60,149]. It is also important to maintain synaptic integrity because synapse loss is strongly associated with cognitive impairment in AD [28]. Phytochemicals can promote synaptic activity in a variety of ways, such as by minimizing oxidative and inflammatory injury, stabilizing mitochondrial energy generation, and augmenting neurotrophic signaling pathways. A number of compounds were found to enhance BDNF expression, which facilitates neuronal survival, dendritic development, and synaptic plasticity [89,100]. The multitarget mode of action of phytochemicals is revealed by their ability to simultaneously affect multiple pathological processes in AD. Most studies, however, are preclinical despite strong laboratory evidence. Before phytochemicals can be fully translated into clinical intervention strategies for the treatment of AD, important issues such as poor bioavailability, limited penetration across the BBB, and a lack of large-scale clinical trials should be addressed [65].

## 6. Multi-Target Neuroprotective Mechanisms of Phytochemicals in Alzheimer’s Disease

Phytochemicals are also gaining recognition for their ability to support neuronal survival and functional stability at various stages of AD. In contrast to single-target pharmacological agents, most plant-derived compounds exhibit broad biological actions that enhance neuronal resilience against chronic pathological conditions. One of these conditions is AD, a degenerative process that evolves progressively in a complex process involving oxidative imbalance, neuroinflammation, mitochondrial dysfunction, proteostatic impairment, and progressive synaptic loss. Phytochemicals may interrupt this cascade by strengthening cellular defense mechanisms and preserving neuronal homeostasis before irreversible structural damage occurs. This concept is particularly relevant because AD pathology can begin 10–20 years before clinical symptoms emerge, providing a long window for preventive interventions that enhance neuronal resilience [150,151].

**Figure 4:**
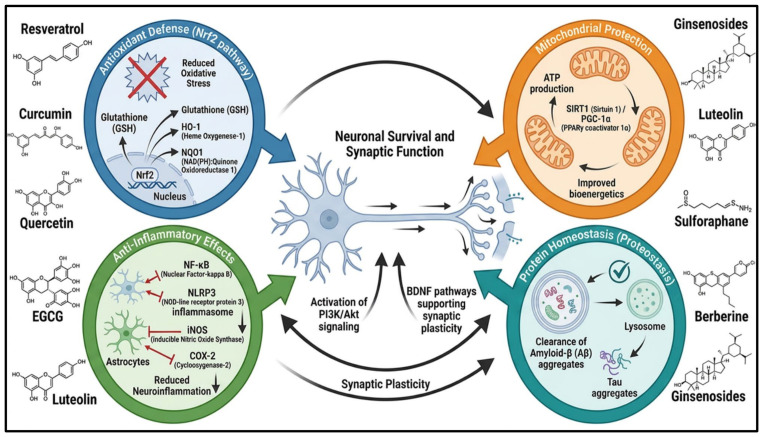
Multi-Target Molecular Mechanisms of Phytochemical-Mediated Neuroprotection in Alzheimer’s Disease: Regulation of Oxidative Stress, Neuroinflammation, Mitochondrial Function, Proteostasis, and Pro-Survival Signaling Pathways.

Figure 4 shows a comprehensive overview of the multi-target neuroprotective actions of phytochemicals against AD pathology via antioxidant, anti-inflammatory, mitochondrial, proteostatic, and neurotrophic signaling pathways. Representative phytochemicals, including curcumin, resveratrol, quercetin, EGCG, berberine, luteolin, ginsenosides, and sulforaphane, exert neuroprotective effects by simultaneously modulating multiple interconnected molecular pathways involved in AD [152,153]. These compounds enhance cellular antioxidant defenses by activating the Nrf2/HO-1 pathway and upregulating antioxidant enzymes, including superoxide dismutase (SOD), catalase (CAT), and glutathione peroxidase (GPx), thereby reducing reactive oxygen species (ROS). They suppress neuroinflammation by inhibiting NF-κB signaling and NLRP3 inflammasome activation, thereby reducing the production of pro-inflammatory cytokines, including tumor necrosis factor-α (TNF-α) and interleukin-6 (IL-6). Phytochemicals also preserve mitochondrial integrity by improving ATP production, reducing mitochondrial ROS generation, and maintaining mitochondrial membrane potential. In parallel, they restore proteostasis by promoting autophagy–lysosomal degradation and ubiquitin–proteasome-mediated clearance of misfolded proteins, facilitating the removal of amyloid-β and hyperphosphorylated tau aggregates. Furthermore, activation of PI3K/Akt, BDNF, SIRT1, and related pro-survival pathways enhances neuronal survival, synaptic plasticity, and cognitive function, collectively attenuating AD progression.

As summarized in Table 5, the level of evidence supporting neuroprotective phytochemicals in AD varies substantially across experimental and clinical stages. Most compounds, including EGCG, quercetin, berberine, luteolin, ginsenoside Rg1, sulforaphane, astaxanthin, oleocanthal, urolithin A, apigenin, naringenin, fisetin, baicalein, ferulic acid, kaempferol, withanoside IV/sominone, and bacoside A, are primarily supported by in vitro and animal-model evidence, indicating strong mechanistic potential but limited clinical translation. In contrast, curcumin, resveratrol, huperzine A, genistein, saffron-derived compounds, and macular carotenoids have progressed to early-phase or pilot clinical evaluation, although findings remain mixed, preliminary, or insufficient to draw definitive therapeutic conclusions. Galantamine, included as a plant-derived reference compound, remains the only phytochemical-related agent in the table with robust clinical evidence and established symptomatic use in AD. Therefore, Table 5 highlights the need for well-designed, adequately powered randomized controlled trials to validate the translational potential of phytochemical-based interventions in AD.

### 6.1. Preservation of Neuronal Homeostasis

One of the neuroprotective effects of phytochemicals in AD is the maintenance of neuronal homeostasis under chronic cellular stress. Neuronal viability depends on a closely controlled redox balance, mitochondrial ATP production, calcium signalling, and protein quality-control mechanisms. These regulatory mechanisms are increasingly impaired in AD by amyloid-β toxicity, tau pathology, oxidative damage, and inflammatory signaling, and eventually lead to impaired neuronal survival. Most phytochemicals increase neuronal resistance by stimulating endogenous defense systems rather than acting as direct antioxidants. Stated differently, a number of compounds such as sulforaphane, curcumin, resveratrol, and several flavonoids activate the Nrf2 signaling pathway, which controls the expression of the antioxidant and cytoprotective enzymes such as heme oxygenase-1, glutathione-related enzymes, and NAD(P)H quinone oxidoreductase-1. Activation of these pathways strengthens cellular defense capacity and improves neuronal tolerance to oxidative stress [33,60].

The maintenance of mitochondrial function is also an important aspect of neuronal protection. Because neurons require continuous ATP production for neurotransmission, axonal transport, and calcium buffering, mitochondrial impairment plays a central role in AD progression. Experimental studies indicate that several phytochemicals can stabilize mitochondrial membrane potential, reduce mitochondrial oxidative stress, and improve bioenergetic efficiency. For instance, resveratrol has been associated with activation of the SIRT1/PGC-1α pathway, which is involved in mitochondrial biogenesis, while compounds such as ginsenosides, astaxanthin, and sulforaphane have demonstrated beneficial effects on mitochondrial redox balance and energy metabolism in experimental models [57,100,154].

Proteins are also involved in neuronal homeostasis. In AD, defective autophagy and proteasomal degradation pathways increase the aggregation of misfolded proteins, including amyloid-β and hyperphosphorylated tau. It has been reported that several phytochemicals, such as berberine, curcumin, resveratrol, and sulforaphane, can promote autophagic flux, increase lysosomal degradation, and clear toxic intracellular aggregates [38,155]. In addition, phytochemicals can modulate neuroinflammatory responses. Chronic activation of microglia and astrocytes produces pro-inflammatory cytokines and RNS that exacerbate oxidative stress and synaptic injury. Compounds such as luteolin, quercetin, curcumin, and sulforaphane have demonstrated the ability to suppress inflammatory mediators, including NF-κB, NLRP3, iNOS, and cyclooxygenase-2 (COX-2), thereby reducing inflammatory amplification within the central nervous system [21,37,87].

### 6.2. Maintenance of Synaptic Function and Pro-Survival Signaling

One of the most significant determinants of cognitive functioning in AD is preservation of synaptic integrity. Clinical and pathological research demonstrates that neuronal connectivity and synaptic communication, which are associated with cognitive decline, are linked to synapse loss rather than to amyloid plaque burden alone [16,28]. Phytochemicals can facilitate synaptic stability by alleviating upstream toxic stress and stimulating intracellular survival pathways. Synaptotoxic effects of soluble Aβ oligomers, inhibiting LTP, disrupting calcium homeostasis, and changing the structure of dendritic spines. There are a number of phytochemicals, such as EGCG, curcumin, resveratrol, and quercetin, that have been shown to reduce amyloid aggregation, down-regulate tau pathology, and counteract the synaptotoxic effects of amyloid oligomers of soluble proteins [31,48].

Phytochemicals influence signaling pathways, including Nrf2 (antioxidant response), NF-κB (inflammatory signaling), and PI3K/Akt (neuronal survival), thereby enhancing neuroprotection. Different phytochemicals have been shown to activate this pathway, thereby promoting neuronal survival and reducing neurodegenerative damage in animal models [49,156]. Other phytochemicals also enhance BDNF signaling, which facilitates neuronal survival, dendritic plasticity, and synaptic plasticity in memory formation [157,158]. These protective effects are also explained by the concept of phytochemical neurohormesis. This model posits that some phytochemicals induce mild adaptive stress responses that enhance the cellular defense system and make neurons tolerant to subsequent pathological insults [159]. These adaptive responses can help maintain neuronal function in the long-term during aging and neurodegenerative disease.

This network-based perspective of neuroprotection is supported by evidence from experimental models. For example, quercetin has been shown to reduce AD pathology and enhance cognitive function in transgenic models, whereas berberine has been shown to reduce amyloid deposition, tau pathology, cerebral blood flow, and autophagic clearance [48]. Likewise, astaxanthin has corrected cognitive impairment in APP/PS1 mice, and ginsenoside Rg1 has demonstrated multipurpose neuroprotective properties in AD-like models [160,161]. In other neurodegenerative models, sulforaphane has also demonstrated both consistent cytoprotective and antioxidant activities [37,162].

Despite these encouraging results, significant translational issues remain. A significant part of the existing data is based on cellular and animal research, and well-designed human clinical trials remain scarce. Moreover, most phytochemicals have low oral bioavailability, undergo limited metabolism, and exhibit low brain penetration, making them challenging to use in clinical practice [163,164]. Therefore, although phytochemicals are biologically viable multi-target neuroprotective agents, they require additional studies to determine their therapeutic potential and to improve methods for clinical translation to AD.

### 6.3. Major Phytochemicals and Their Therapeutic Mechanisms in Alzheimer’s Disease

Natural phytochemicals are promising therapeutic agents for AD. They exhibit multiple biological activities, including antioxidant, anti-inflammatory, anti-amyloid, and neuroprotective effects. These properties help target several pathological processes in AD, including amyloid-β aggregation, tau phosphorylation, oxidative stress, mitochondrial dysfunction, and neuroinflammation. Curcumin, a compound from *Curcuma longa*, is one of the most widely studied phytochemicals. It can inhibit amyloid-β aggregation, reduce oxidative stress, and suppress neuroinflammation. These effects occur partly through activation of the peroxisome proliferator-activated receptor gamma (PPAR-γ) signaling pathway. Experimental and clinical studies also suggest that curcumin may improve amyloid pathology and cognitive function [165,166,167]. This clinical evidence must, however, be interpreted with caution. The most frequently cited supporting human study [120] was conducted in non-demented older adults rather than in patients with AD, and a 24-week randomised, double-blind, placebo-controlled trial of oral curcumin in patients with AD [43] found no significant clinical or biomarker effects. Thus, while preclinical and pilot data are encouraging, current human evidence does not establish a disease-modifying benefit of curcumin in AD, and the supportive findings reflect mechanistic and small early-phase studies rather than adequately powered randomised controlled trials.

**Figure 5:**
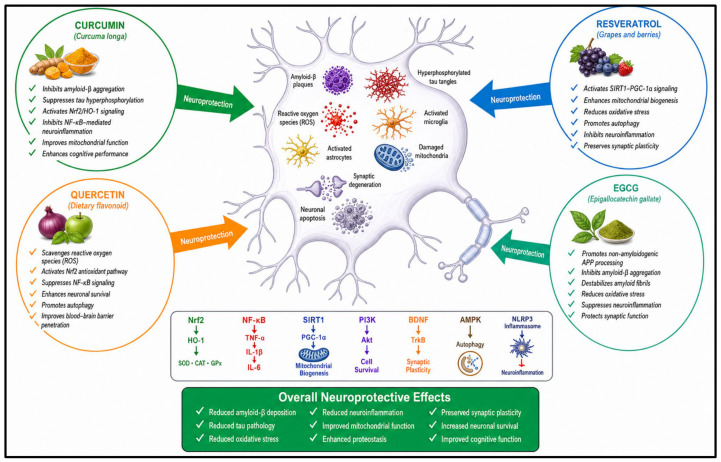
Therapeutic Potential and Multi-Target Neuroprotective Mechanisms of Major Phytochemicals in Alzheimer’s Disease.

Major phytochemicals exhibit considerable therapeutic potential in AD by simultaneously targeting multiple interconnected pathological mechanisms involved in disease progression, as seen in Figure 5. Curcumin inhibits amyloid-β aggregation, attenuates oxidative stress through activation of the Nrf2/HO-1 antioxidant pathway, suppresses NF-κB-mediated neuroinflammation, and improves cognitive function. Resveratrol enhances mitochondrial biogenesis and cellular energy metabolism by activating the SIRT1/PGC-1α signaling pathway, while reducing oxidative stress and preserving synaptic integrity. Quercetin exerts potent antioxidant and anti-inflammatory effects by scavenging reactive oxygen species (ROS), activating endogenous antioxidant defenses, and promoting neuronal survival. EGCG facilitates non-amyloidogenic amyloid precursor protein (APP) processing, inhibits amyloid-β aggregation, destabilizes mature amyloid fibrils, and suppresses neuroinflammatory responses. Collectively, these phytochemicals regulate multiple molecular pathways associated with oxidative stress, mitochondrial dysfunction, protein aggregation, neuroinflammation, and synaptic impairment, thereby preserving neuronal function and slowing neurodegenerative processes. Their pleiotropic mechanisms of action support the development of phytochemical-based multi-target therapeutic strategies for the prevention and management of AD.

On the same note, resveratrol, a polyphenolic antioxidant in grapes and berries, has neuroprotective effects due to its activation of the Sirtuin 1 signaling pathway, which controls mitochondrial activity, oxidative stress, and neuronal survival, and clinical studies have associated resveratrol with positive safety and regulation of biomarkers of AD [168,169]. The neuroprotective potential of quercetin, a widely distributed dietary flavonoid, has also been demonstrated as an ROS scavenger and an inhibitor of neuroinflammatory pathways, with a positive effect on neuronal survival and cognitive performance in experimental (exclusively preclinical) models of AD. Recent studies have aimed to enhance the brain bioavailability of quercetin by nano-delivery [170,171,172]. Moreover, EGCG, the most prominent catechin in green tea, promotes non-amyloidogenic APP processing, reduces amyloid-β protein production, reconfigures toxic fibrils into less toxic forms, and exerts potent antioxidant and anti-inflammatory effects that help preserve neurons [173,174]. Together, these phytochemicals have demonstrated extensive therapeutic potential and the capacity to regulate numerous molecular targets involved in the pathogenesis of AD; a brief description of their actions and experimental results is tabulated in Table 6.

Beyond the core compounds, several additional phytochemicals broaden the therapeutic landscape of AD, although their evidence remains largely preclinical. In 3xTg-AD mice, luteolin improved learning and memory by reducing endoplasmic reticulum stress-dependent neuroinflammation, and complementary AD models further showed protection against Aβ-induced oxidative stress and mitochondrial impairment [35,72]. Quercetin reduced β-amyloid deposition, tau pathology, astrogliosis, and microgliosis while improving cognitive and emotional outcomes in aged 3xTg-AD mice [48]. Berberine improved cognition and reduced Aβ pathology, gliosis, tau hyperphosphorylation, and impaired autophagic clearance in transgenic AD mouse models [190,191]. Among terpenoid and carotenoid compounds, ginsenoside Rg1 restored PINK1/Parkin-mediated mitophagy, reduced hippocampal Aβ deposition, and improved memory in 5xFAD mice, whereas astaxanthin and DHA-acylated astaxanthin improved cognitive deficits and reduced oxidative stress, tau hyperphosphorylation, neuroinflammation, or synaptic injury in APP/PS1 mice [78]. Sulforaphane also reduced Aβ and tau burden in 3xTg-AD mice, consistent with the broader AD-relevant role of Nrf2/ARE signaling in suppressing BACE1 expression and cognitive impairment [71,93]. Other flavonoids and polyphenol-rich extracts, including apigenin, kaempferol, baicalein, and anthocyanin-rich blueberry extracts, have shown AD-relevant benefits in cellular or animal models by reducing Aβ toxicity, oxidative stress, neuroinflammation, behavioural impairment, or autophagy–lysosomal dysfunction [69,73,192]. Collectively, these examples demonstrate that phytochemicals can modulate multiple AD-related mechanisms, but they also emphasize that most evidence remains compound-specific and preclinical; therefore, translation to clinical utility requires cautious interpretation and well-designed human trials. For reference, the chemical identity of the representative phytochemicals discussed throughout this review is summarized in Table 7; their full chemical structures are shown in the regenerated figures.

To distinguish experimentally promising phytochemicals from compounds with validated clinical relevance, the available evidence is summarized in Table 8. Overall, the table shows that only a limited number of phytochemical or phytochemical-derived interventions have been evaluated directly in AD patients, including galantamine, huperzine A, curcumin, resveratrol, saffron extract, genistein, macular carotenoids, *Melissa officinalis*, *Salvia officinalis*, and *Ginkgo biloba* extracts. Among these, galantamine remains the only plant-derived compound with established clinical use as an approved symptomatic AD therapy, whereas resveratrol, curcumin, saffron, genistein, and others.

## 7. Evidence for Phytochemicals from In Vitro, Animal, and Human Studies

Most evidence for neuroprotective efficacy comes from in vitro and animal models. While some compounds, such as resveratrol and curcumin, have been tested in small-scale clinical trials, results remain inconclusive due to low bioavailability, insufficient BBB penetration, and limited sample sizes. An accumulating body of experimental and clinical studies has provided substantial insights into the mechanisms and pathogenesis of AD. In vitro studies based on induced pluripotent stem cell (iPSC)-derived neurons, three-dimensional cultures of the human brain, and cerebral organoids have provided evidence of the occurrence of key pathological hallmarks with amyloid-β accumulation, tau hyperphosphorylation, and neuroinflammatory reactions in cellular models of the human brain [201,202,203]. In the same vein, animal models, especially transgenic rodent models in which APP, PSEN, and tau mutations are expressed, have been crucial in studies of disease pathogenesis since they recapitulate the process of amyloid plaque formation, neurofibrillary tangles, loss of synapses, and cognitive impairment [204,205]. Moreover, clinical trials in humans have tested a wide range of therapeutic approaches, including cholinesterase inhibitors, NMDA receptor blockers, and disease-modifying monoclonal antibodies targeting amyloid-β pathology. Although, the early interventions mainly offered a symptomatic relief effect [206,207].

Recent anti-amyloid antibodies such as lecanemab and donanemab have demonstrated statistically significant but modest slowing of decline in selected early AD populations, while requiring monitoring for amyloid-related imaging abnormalities. Aducanumab warrants particularly cautious interpretation. It received accelerated approval from the US FDA in 2021 on the basis of amyloid-plaque reduction, in a decision that was itself widely contested because the two pivotal Phase 3 trials (EMERGE and ENGAGE) yielded discordant results and an independent advisory committee had recommended against approval. Owing to limited clinical uptake and persistent questions over clinical benefit, Biogen subsequently withdrew the marketing applications and discontinued development of the antibody in 2024. Accordingly, the early-phase positive data shown in Table 9 should be read alongside these later Phase 3 failures and the eventual discontinuation, rather than as evidence of validated clinical efficacy [208,209,210].

All these experimental, animal, and clinical studies demonstrate that AD is a complex and multifactorial neurodegenerative disorder involving amyloid-β accumulation, tau pathology, neuroinflammation, synaptic dysfunction, and genetic risk factors. Experimental studies using iPSC-derived neurons, cerebral organoids, and 3D neural cultures have provided important insights into disease mechanisms, as summarized in Table 9. Similarly, animal studies have explained amyloid and tau propagation, neurodegeneration, and microglial involvement in AD pathogenesis (Table 9a). Furthermore, clinical trials have evaluated both symptomatic and disease-modifying therapies, highlighting the challenges and recent progress in AD treatment development (Table 9b). Together, these findings provide strong evidence supporting current understanding and therapeutic strategies for AD.

## 8. Current Challenges, Limitations, and Research Gaps in Phytochemical-Based Therapies for Alzheimer’s Disease

### 8.1. Pharmacokinetic, Delivery, and Clinical Translation Challenges

**Figure 6:**
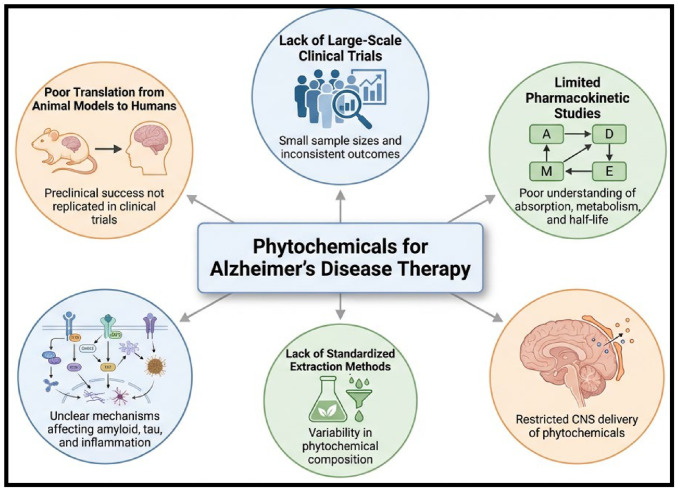
Key Research Gaps Limiting the Clinical Translation of Phytochemicals for Alzheimer’s disease.

Although phytochemicals have demonstrated considerable neuroprotective potential in experimental models of AD, several important limitations continue to restrict their successful translation into clinical therapeutics, as illustrated in Figure 6. These challenges primarily involve poor pharmacokinetic properties, limited BBB permeability, inadequate clinical validation, and insufficient standardization of therapeutic formulations. Despite extensive evidence supporting the antioxidant, anti-inflammatory, and anti-amyloidogenic effects of phytochemicals such as curcumin, resveratrol, flavonoids, and ginsenosides, only a limited number of large-scale randomized clinical trials have evaluated their efficacy in human populations. Most available studies are characterized by small sample sizes, short intervention durations, and heterogeneous outcome measures, making it difficult to establish definitive therapeutic conclusions [236]. A major obstacle is the inadequate pharmacokinetic characterization of phytochemicals. Important absorption, distribution, metabolism, and excretion (ADME) parameters remain poorly understood for many plant-derived compounds, thereby limiting dose optimization and therapeutic standardization. For instance, curcumin and resveratrol undergo rapid hepatic and gastrointestinal metabolism, resulting in extremely low systemic bioavailability and reduced concentrations of active metabolites in circulation [237,238]. Furthermore, the inability of many phytochemicals to efficiently cross the BBB significantly compromises their therapeutic effectiveness in targeting central nervous system pathology associated with AD [239]. Although some compounds demonstrate partial BBB permeability, systematic investigations into their transport mechanisms and brain bioavailability remain limited. Advanced drug-delivery strategies, including nanoparticle-based carriers, liposomal formulations, and lipid-based delivery systems, have shown promise for enhancing CNS delivery; however, these approaches remain underexplored in clinical settings.

In addition to pharmacokinetic limitations, safety, regulatory, and quality-control issues further complicate the clinical application of phytochemicals. Variability in phytochemical composition arising from differences in plant species, environmental conditions, harvest periods, and extraction methods contributes to inconsistent therapeutic outcomes. The absence of standardized extraction and characterization procedures reduces reproducibility across studies and limits regulatory approval of phytochemical-based formulations [240]. For example, preparations of *Curcuma longa* and *Ginkgo biloba* may contain significantly different concentrations of active constituents depending on processing and manufacturing practices [241]. Consequently, the development of universally accepted quality-control guidelines remains a critical requirement for future phytochemical therapeutics.

### 8.2. Mechanistic Gaps, Preventive Research, and Future Directions

**Figure 7:**
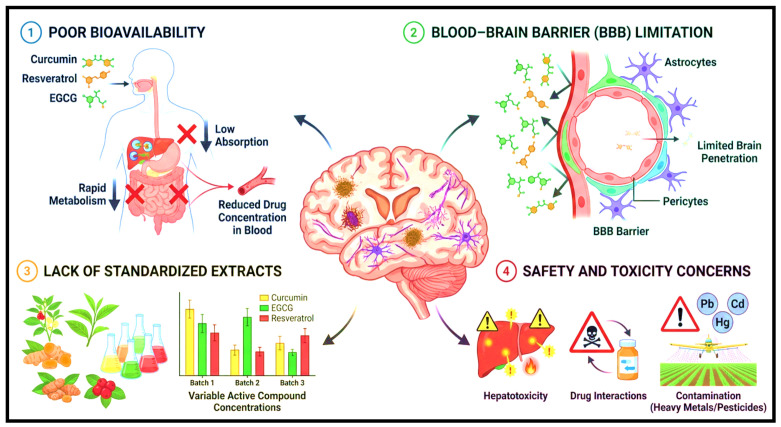
Challenges Limiting the Clinical Translation of Phytochemicals for Alzheimer’s Disease Therapy.

Beyond pharmacokinetic and formulation-related limitations, several mechanistic and translational research gaps continue to hinder progress in phytochemical-based AD therapeutics. AD is a highly multifactorial neurodegenerative disorder involving amyloid-β aggregation, tau hyperphosphorylation, oxidative stress, mitochondrial dysfunction, synaptic impairment, and neuroinflammation. Although numerous phytochemicals exhibit neuroprotective activities against these pathological processes, the precise molecular targets and signaling pathways responsible for their therapeutic actions remain incompletely understood. As highlighted in Figure 7, limited mechanistic understanding restricts the rational development of targeted phytochemical therapies [242]. Current research increasingly emphasizes the importance of advanced omics technologies, including proteomics, transcriptomics, metabolomics, and systems biology, for identifying the specific molecular pathways influenced by phytochemicals. These approaches may facilitate the discovery of novel therapeutic targets and improve understanding of synergistic interactions among multiple phytochemical constituents. Moreover, there remains a substantial lack of long-term preventive studies evaluating whether sustained dietary intake or nutraceutical supplementation with phytochemicals can delay the onset or progression of AD. Since AD pathology may begin decades before clinical symptoms become apparent, long-term longitudinal studies are essential to determine the preventive efficacy of phytochemical interventions against cognitive decline [243]. Another major challenge involves the poor translation of findings from animal models to human clinical outcomes. Although transgenic mouse models have substantially contributed to understanding AD pathogenesis, they fail to fully replicate the complexity and heterogeneity of human disease pathology. Consequently, many phytochemicals that demonstrate promising neuroprotective effects in preclinical studies fail to exhibit similar efficacy in human clinical trials [244]. Addressing this translational gap will require developing more representative experimental systems, including human-induced pluripotent stem cell models, brain organoids, and advanced computational disease models. Integrating artificial intelligence, systems pharmacology, and precision medicine approaches may further enhance the predictive accuracy of preclinical investigations and accelerate the clinical development of phytochemical-based therapeutics.

### 8.3. Poor Bioavailability and Blood–Brain Barrier Penetration

A major issue with phytochemicals is their low bioavailability, which reduces their therapeutic value. Many plant-derived compounds, particularly poorly water-soluble polyphenols and flavonoids, have limited gastrointestinal absorption and undergo extensive first-pass metabolism. These substances are frequently subject to high first-pass metabolism in the intestines and liver following oral administration, which substantially decreases their systemic availability [245,246]. For example, the phytochemical curcumin, which is widely investigated for its effects on neurodegenerative diseases, has excellent anti-inflammatory and antioxidant effects but exhibits the lowest plasma levels due to low absorption and high metabolism [247]. In the same case, the resveratrol and EGCG are rapidly metabolized into inactive metabolites that curtail their therapeutic effects in vivo [248]. It has therefore been suggested that different strategies could be used to increase the bioavailability of phytochemicals and improve their pharmacological effects through nanoparticle delivery systems, liposomal formulations, and structural modifications [249].

Another significant constraint is that many phytochemicals have a limited capacity to cross the BBB. The BBB is a highly selective biological barrier that controls what enters the central nervous system. Many phytochemicals possess molecular characteristics, such as high polarity or large molecular size, that prevent their transport across the BBB [250]. Consequently, although such compounds may exhibit strong neuroprotective effects in vitro, their brain penetration can be limited due to poor penetration through neural tissue. Research studies have indicated that compounds such as curcumin and resveratrol have low brain bioavailability despite their high anti-amyloid and anti-inflammatory activity [251]. To address this shortcoming, researchers are considering new approaches to drug delivery, including nanocarriers, lipid-based delivery systems, and intranasal delivery, which could improve drug delivery across the BBB and enhance neuroprotective outcomes [252].

### 8.4. Lack of Standardized Extracts, Safety, and Toxicity Concerns

Another important issue in phytochemical studies is the lack of standardized plant extracts. Plant-derived extracts may vary widely in chemical composition depending on plant species, geographic origin, climate, harvest time, and extraction method [253]. Such variability might lead to uneven concentrations of active substances, and hence the results of different studies may not be reproducible. For example, an herbal preparation containing Ginkgo biloba or Curcuma longa can differ significantly in the levels of bioactive compounds such as flavonoids or curcuminoids [246]. In the absence of standardization, determining the accurate therapeutic dose is difficult, as is comparing the results of experimental and clinical studies. Thus, it is necessary to establish standard extraction procedures, quality control protocols, and chemical fingerprints to enhance the reliability and reproducibility of phytochemical studies.

Although many phytochemicals are widely consumed in foods or herbal products, natural origin does not guarantee safety, especially at concentrated doses or during long-term use. To illustrate, some flavonoids and alkaloids in large amounts are linked to hepatotoxicity, gastrointestinal problems, and drug interactions with conventional ones [254]. Moreover, most of the phytochemicals have not been evaluated in long-term safety-in-clinic evaluations, especially among the elderly who might already be taking several drugs. Moreover, herbal preparations can change, thereby increasing the risk of contamination with pesticides, heavy metals, or adulterants [253]. Hence, rigorous toxicological research and well-designed clinical trials are needed to ensure the safety, efficacy, and appropriate dosing of phytochemical-based treatments for AD.

## 9. Emerging Strategies to Improve Phytochemical Therapy

Although the neuroprotective effect of phytochemicals in animal models of AD is advocated, their application in clinical treatments has not been effective. Various obstacles are associated with their therapeutic use, including limited solubility, low bioavailability, rapid metabolism, limited BBB permeation, and variability in plant extract composition. As a result, recent studies have shifted toward developing sophisticated measures to enhance the delivery, efficacy, and clinical applicability of phytochemical-based therapies. These are nanotechnology-based drug delivery systems, combination therapy with traditional drugs, multi-target drug design, and artificial intelligence (AI)-based drug discovery [65,156,163].

### 9.1. Nanotechnology-Based Delivery Systems

The use of nanotechnology-based drug-delivery systems has emerged as a promising approach to enhance the therapeutic effectiveness of neuroprotective compounds in AD. The existence of the BBB, a highly selective physiological barrier that limits the entry of most therapeutic agents into the central nervous system, is a key issue in the development of effective treatments for AD [250,255]. Thus, a variety of potentially useful compounds, such as phytochemicals with proven neuroprotective activity, have limited clinical potential due to low bioavailability, rapid metabolism, and lack of BBB penetration. Nanotechnology can provide novel solutions to these restrictions by enabling targeted delivery of therapeutic molecules, controlled release, and improved stability [256,257].

Nanoparticle-based delivery systems (liposomes, polymeric nanoparticles, solid lipid nanoparticles, dendrimers, nanomicelles, and metallic nanoparticles) have demonstrated significant capacity to enable drug delivery across the BBB [255,258]. These nanocarriers can entrap bioactive molecules, deliver them to the body without enzymatic degradation, and enhance their pharmacodynamic and pharmacokinetic properties. Besides that, surface-coating nanoparticles with targeting ligands, such as peptides, antibodies, or receptor-specific molecules, may facilitate receptor-mediated endocytosis across the BBB and selective deposition in brain tissues [257,259]. These directed delivery approaches enhance the localization of therapeutic agents to the site of disease and reduce systemic toxicity.

**Figure 8:**
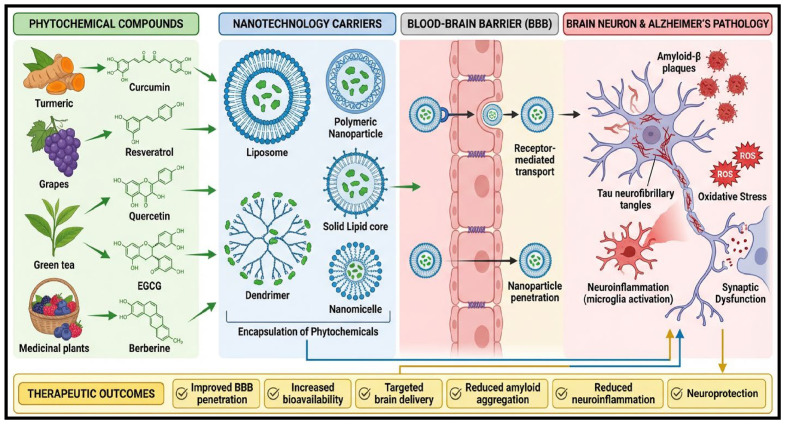
Nanotechnology-Based Delivery of Phytochemicals Across the Blood–Brain Barrier for Targeting Alzheimer’s Disease Pathology.

Nanotechnology has been extensively used in phytochemical-based AD therapy to improve the bioavailability and brain delivery of compounds such as curcumin, resveratrol, quercetin, EGCG, and berberine. Nanoformulations of the compounds have been shown to exhibit improved solubility, greater BBB permeability, extended circulation, and enhanced neuroprotective effects in test models [260,261]. To illustrate, nanoformulations of curcumin have been found to inhibit amyloid-β aggregation and neuroinflammation more efficiently, and resveratrol nanoformulations have been shown to exhibit antioxidant properties in neuronal cells [262,263]. Figure 8 depicts that phytochemical molecules of medicinal plants may be loaded in a variety of nanoparticles, including liposomes, polymeric nanoparticles, dendrimers, solid lipid nanoparticles, and nanomicelles, in order to deliver their cargo across the BBB and to specific pathological processes implicated in the development of AD, including amyloid-β plaque formation, tau neurofibrillary tangles, oxidative damage, neuroinflammation, and synaptic dysfunction.

In addition to enhancing drug delivery, nanotechnology can be used to create multifunctional nanotherapeutics that target numerous pathological processes contributing to AD. Other nanoparticle systems are engineered to carry both therapeutic agents and diagnostic probes, thus facilitating theranostic applications that integrate treatment and disease monitoring [264]. Other engineered nanocarriers can be designed to respond to specific physiological stimuli, e.g., pH, oxidative stress, or the enzymatic activity of the diseased brain microenvironment, and to release drugs under control and at the site [265,266]. These strategies could enable a more accurate regulation of the primary AD-related processes, such as amyloid deposition, tau pathology, neuroinflammation, and mitochondrial dysfunction.

Notwithstanding these promising developments, several issues remain to be addressed before nanotechnology-based therapies become a common part of clinical practice. The concerns about the long-term safety and toxicity of nanoparticles, large-scale production, regulatory status, and the reliability of nanoformulations are to be addressed [267,268]. Moreover, less work has been done on translating experimental models into human clinical trials, and there is a call for a thorough analysis of the pharmacokinetics, biodistribution, and therapeutic effectiveness in well-designed clinical trials.

### 9.2. Combination Therapy with Conventional Drugs

Combinational therapy is an emerging approach that is among the strongest agents for enhancing therapeutic outcomes in AD, especially given the complex, multi-factorial nature of the disease, alongside amyloid-β aggregation, tau hyperphosphorylation, oxidative stress, mitochondrial dysfunction, neuroinflammation, and synaptic degeneration. The majority of conventional pharmacological interventions (e.g., acetylcholinesterase inhibitors, e.g., donepezil, rivastigmine, and galantamine; or N-methyl-D-aspartate, NMDA, receptor antagonist memantine) mainly provide symptomatic relief without effectively stopping the disease progression [269,270]. Therefore, the use of synergistic agents of conventional medications and phytochemicals with multitarget neuroprotective characteristics is becoming a topic of more and more interest as a possible approach to medication. Phytochemicals may be used in combination with available pharmacological therapies to regulate oxidative stress, neuroinflammation, mitochondrial dysfunction, and abnormal protein aggregation that are not controlled by current drugs [156,271].

A number of preclinical and experimental trials have examined the therapeutic value of co-administering phytochemicals with approved drugs. For example, resveratrol, an antioxidant and anti-inflammatory polyphenol, has been shown to synergize with donepezil to reduce the effects of amyloid-β buildup, tau phosphorylation, and cognitive impairment in a mouse model of AD [191,272]. Likewise, curcumin, in its nanoformulation mixed with the donepezil compound, was shown to increase antioxidant defenses, decrease neuroinflammatory signaling, and improve the functioning of behavioral systems in neurodegenerative animal models [273]. There are also synergistic effects between other phytochemicals, such as quercetin, EGCG, and berberine, and conventional drugs that should improve neuroprotective signaling pathways and neuronal survival [274,275].

Despite these encouraging results, careful consideration is needed for the clinical use of phytochemical-drug combinations. Plant-based compounds have the potential to affect the actions of drug-metabolizing enzymes, particularly cytochrome P450 isoforms, thereby altering the pharmacokinetics and therapeutic efficacy of co-administered drugs [276]. As such, rigorous pharmacological and clinical studies are needed to establish the safety, efficacy, and optimal dosing of combination regimens incorporating phytochemicals and traditional drugs. Combination therapy can be a valid approach to enhance the outcomes of long-term therapeutic interventions for AD, and the future involvement of pharmacokinetic studies, biomarker-directed clinical trials, and precision medicine approaches can help establish its effectiveness.

### 9.3. Multi-Target Drug Design

Multi-target therapeutic strategies have been of growing interest in light of the multiple factors and complexity of AD pathophysiology. Classical drug discovery methods have focused more on single molecular targets, such as β-secretase, acetylcholinesterase, or the NMDA receptor. These approaches, however, have been shown to be clinically unsuccessful since AD pathogenesis comprises a variety of interconnected biological events such as amyloid-β aggregation, tau hyperphosphorylation, oxidative stress, mitochondrial dysfunction, chronic neuroinflammation, and synaptic degeneration [242,277,278]. Consequently, a therapeutic intervention that relies solely on a specific pathway can be inadequate and fail to prevent disease progression.

Multi-target drug design has thus become a new approach to designing compounds that can simultaneously regulate multiple disease-related pathways. Multi-target-directed ligands (MTDLs), unlike traditional single-target drugs, are designed to engage multiple molecular targets implicated in neurodegeneration. This practice also reflects the growing recognition that intricate neurological conditions like AD require therapeutic measures that restore network-scale cellular homeostasis rather than targeting specific molecular mechanisms [279,280]. Multi-target options can also simultaneously modulate amyloid processing, tau pathology, the oxidative stress response, inflammatory signaling, and neurotransmitter imbalance, offering broader neuroprotective benefits.

The targets of phytochemicals have shown considerable promise for multi-target mechanism design, as a significant proportion of natural products exhibit pleiotropic pharmacological effects. As an example, various biological pathways that are relevant to AD, such as antioxidant defense mechanisms, neuroinflammatory pathways, mitochondrial physiology, and amyloid aggregation, have been found to be regulated by polyphenolics such as curcumin, resveratrol, and quercetin [271,281]. These compounds can modulate key signaling pathways, including the Nrf2-mediated antioxidant response, the NF-κB inflammatory signaling pathway, and the PI3K/Akt neuronal survival pathway, thereby exerting broad neuroprotective effects.

New breakthroughs in medicinal chemistry have also accelerated the development of hybrid molecules that combine the pharmacophores of known drugs with scaffolds derived from natural molecules. For example, the molecular design of hybrid molecules combining the acetylcholinesterase-inhibitor framework of donepezil has been achieved by incorporating features of flavonoids, polyphenols, or other bioactive phytochemicals. They have shown the potential to coordinate inhibition of cholinesterase activity and amyloid aggregation, as well as to affect oxidative stress and neuronal survival, in experimental models [282,283]. These MTDLs represent a promising approach to AD drug discovery, as they can act within the complex molecular network underlying disease progression.

Despite these developments, there are still problems in the design and clinical translation of multi-target therapeutics. A challenge in developing compounds that modulate multiple pathways effectively without side effects is balancing target selectivity with pharmacokinetic properties and safety profiles. However, computational drug design, systems pharmacology, and the optimization of multi-target compounds using AI are likely to accelerate the discovery and optimization of multi-target compounds for AD [125,284].

### 9.4. Artificial Intelligence-Assisted Drug Discovery

Artificial intelligence (AI) and machine learning (ML) are increasingly used in contemporary drug discovery, particularly for identifying and optimizing phytochemical-based therapeutics for AD. The conventional methods of drug discovery are time-consuming, costly, and high in attrition. Conversely, computational models that are powered by AI allow for the analysis of large quantities of chemical data, predicting drug–target interactions and ranking bioactive compounds with possible neuroprotective activity [285,286]. By combining cheminformatics, molecular simulations, and predictive analytics, AI platforms can screen thousands of plant-derived compounds and identify candidates targeting major molecular pathways implicated in AD pathogenesis.

In studies of phytochemicals, AI-based approaches can help mitigate bias in exploring medicinal plant natural product libraries. ML algorithms can predict the pharmacological properties of phytochemicals based on their structural and physicochemical characteristics, including BBB permeability, target-binding affinity, and pharmacokinetics. These predictive models can also be used to perform early assessments of absorption, distribution, metabolism, excretion, and toxicity (ADMET) profiles, thereby making the lead compound selection process more effective before experimental validation [287]. These computational approaches can greatly increase the speed of initial drug discovery and make it less expensive and more complex than the traditional large-scale screening techniques.

**Figure 9:**
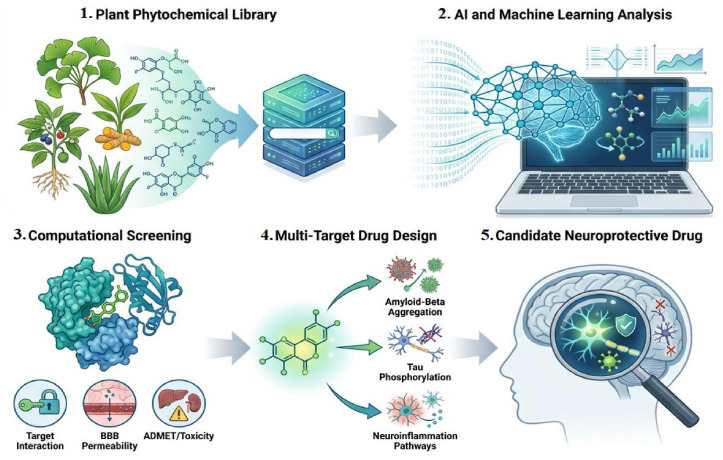
AI-Assisted Screening and Optimization of Phytochemicals Targeting Alzheimer’s Disease Pathways.

As shown in Figure 9**,** AI-assisted drug discovery leverages phytochemical libraries, computational screening, and ML to identify candidate neuroprotective agents that target the underlying pathological mechanisms of AD. The methods enable the logical development of multi-target therapeutic molecules that can simultaneously regulate amyloid-β aggregation, tau hyperphosphorylation, and neuroinflammatory signaling pathways (key pathways in AD development). The use of AI-based approaches, integrating natural product databases with robust predictive models and molecular docking simulations, will enable the creation of a potent framework to accelerate the development and optimization of phytochemical-derived therapeutics as preventive or slowing measures against AD [288].

## 10. Future Perspectives

The growing body of evidence highlighting the neuroprotective properties of phytochemicals suggests that plant-derived compounds may represent promising candidates for the prevention and treatment of AD. However, overcoming current limitations and research gaps requires a multidisciplinary approach that integrates pharmacology, neuroscience, biotechnology, and clinical medicine. Future research should focus on improving the pharmacological properties of phytochemicals, understanding their molecular mechanisms of action, and validating their therapeutic efficacy through well-designed clinical trials.

### 10.1. Advanced Drug Delivery Systems

Among the research areas that promise significant advances are the development of improved drug delivery systems to increase the bioavailability of phytochemicals and brain-targeted delivery systems. Nanoparticles and liposomes, solid lipid nanoparticles, and polymeric micelles are nanotechnology-based delivery platforms that portend to enhance the stability, solubility, and BBB penetration of natural products [289]. These systems can prevent degradation of phytochemicals by metabolic processes and regulate drug release, thereby improving their therapeutic effectiveness. For example, nanoformulations of curcumin and resveratrol have been shown to be more effective at delivering these compounds to the brain and exerting neuroprotective effects against neurodegeneration in experimental models.

### 10.2. Multi-Target Therapeutic Approaches

AD is a complex multifactorial disease having complicated pathological mechanisms such as amyloid-β aggregation, tau hyperphosphorylation, oxidative stress, mitochondrial dysfunction, neuroinflammation, and loss of synapses. Consequently, future therapeutic strategies must adopt multi-target approaches that simultaneously alter multiple pathological processes. In that regard, phytochemicals are of particular interest, as many plant-derived natural products exhibit numerous biological effects, including antioxidant, anti-inflammatory, anti-amyloidogenic, and neuroprotective effects [290]. Exploration of phytochemical combinations, or their combination with existing pharmacological treatments, can have a synergistic effect in delaying disease development.

### 10.3. Integration of Omics and Systems Biology

Recent developments in genomics, proteomics, metabolomics, and systems biology offer robust methods for determining the molecular targets and signaling pathways affected by phytochemicals. Such technologies can be used to elucidate the effects of plant-derived compounds on cellular networks mediating neurodegeneration. Examples include transcriptomic and proteomic profiling, which can reveal how phytochemicals alter gene expression in neuronal cells, and metabolomics, which can identify metabolic pathways that these compounds alter. Combining these methods with computational modeling could help identify new therapeutic targets and design phytochemical-based interventions in the most efficient way.

### 10.4. Development of Standardized Herbal Formulations

The other crucial line of activity for the future is advancing standardized herbal preparations with a clear chemical composition and stable therapeutic activity. Standardization should be implemented to achieve reproducibility in both experimental and clinical research. The development of chemical fingerprints of plant extracts and the identification of their active constituents, which are involved in therapeutic effects, may be facilitated by advances in analytical tools and methods, including high-performance liquid chromatography (HPLC), mass spectrometry, and metabolomic profiling [253]. The reliability and safety of phytochemical-based therapies will be enhanced by developing international standards for herbal medicines and implementing quality control measures.

### 10.5. Long-Term Preventive Studies

Long-term dietary patterns rich in fruits, vegetables, herbs, and spices may provide sustained exposure to diverse phytochemicals, but their preventive effects require confirmation through longitudinal cohort studies and randomized dietary interventions. Such studies could clarify whether phytochemical-rich diets reduce the risk or delay the progression of cognitive decline. Dietary phytochemicals could also help protect the brain by inhibiting oxidative stress and inflammation. To support the hypothesis that routine intake of diets rich in phytochemicals can slow the development or progression of AD, large-scale epidemiological studies and long-term clinical trials are required [291]. They may be useful in preventing cancer and other diseases, as such investigations may provide valuable evidence for the adoption of phytochemicals.

### 10.6. Improved Translational Models

These models can provide more human-relevant insights into neurodegenerative mechanisms and improve preclinical prediction of therapeutic efficacy. Animal models do not necessarily recapitulate the complexity of human AD, which may explain why therapeutic candidates perform poorly in clinical trials [292]. New technologies, including human brain organoids, induced pluripotent stem cell-derived neuronal models, and sophisticated computational simulations, have the potential to replace the use of animals in disease pathology research and in preclinical trials of novel therapies. These models can offer better insights into how humans degenerate their brains and can hasten the development of successful therapies.

## 11. Conclusions

Phytochemicals represent mechanistically promising multitarget agents for AD, capable of modulating interconnected pathological processes, including oxidative stress, neuroinflammation, amyloid-β aggregation, tau hyperphosphorylation, mitochondrial dysfunction, and synaptic degeneration. Compounds including curcumin, resveratrol, EGCG, quercetin, berberine, ginsenosides, luteolin, and sulforaphane demonstrate pleiotropic neuroprotective effects in preclinical models, highlighting their potential to restore cellular homeostasis and network-level neuronal function. These multitarget properties make phytochemicals a compelling approach compared with conventional single-target pharmacological strategies. Despite strong mechanistic evidence in cell and animal models, clinical translation remains limited. Challenges include low oral bioavailability, insufficient penetration of the blood–brain barrier, metabolic instability, variability in phytochemical composition, and a scarcity of adequately powered, biomarker-guided human trials. Consequently, most evidence remains preclinical, and the therapeutic efficacy of phytochemicals in patients with AD has yet to be fully validated. Future research should focus on overcoming these translational barriers through standardized formulations, nanocarriers, or other advanced delivery systems to enhance brain bioavailability and rigorous, biomarker-informed clinical trials to evaluate safety and efficacy. Integrating these strategies may enable phytochemical-based interventions to move from preclinical promise to clinically meaningful prevention and treatment of AD, offering a scientifically grounded, multitarget approach to a complex neurodegenerative disease.

## Figures and Tables

**Table 1:** Core Pathophysiological Mechanisms Underlying Neurodegenerative Diseases and Their Relevance to Phytochemical-Based Neuroprotection.

Pathophysiological Process	Key Molecular Events	Cellular/Molecular Consequences	Major Neurodegenerative Diseases	Therapeutic Relevance for Phytochemicals	Citations	Strength of Evidence
Oxidative Stress	Excessive production of reactive oxygen species (ROS), including superoxide (O_2_^•−^), hydrogen peroxide (H_2_O_2_), hydroxyl radicals (^•^OH), and singlet oxygen, is generated primarily from mitochondrial electron transport chain dysfunction and oxidase enzymes	Lipid peroxidation, protein oxidation, DNA damage, mitochondrial injury, impaired redox signaling, and activation of stress-response pathways (e.g., Nrf2, MAPK)	AD, PD, HD, ALS	Phytochemicals may modulate oxidative stress via antioxidant activity, ROS scavenging, Nrf2 activation, metal chelation, and mitochondrial stabilization	[18]	Strong
Oxidative Stress–Induced Neuronal Injury	Oxidative modification of membrane lipids, mitochondrial proteins, and nucleic acids	Synaptic dysfunction, mitochondrial depolarization, activation of apoptotic pathways, neuronal senescence, and neurodegeneration	AD, PD, ALS, HD	Natural compounds with antioxidant and anti-inflammatory properties may attenuate oxidative damage and delay neuronal degeneration	[19]	Strong
Neuroinflammation	Microglial activation triggered by protein aggregates, ROS, and neuronal injury	Chronic inflammatory signaling, impaired phagocytosis, propagation of protein aggregates, and neurodegenerative progression	AD, PD, ALS, HD, FTD	Phytochemicals may regulate microglial activation and suppress inflammatory signaling pathways such as NF-κB, NLRP3 inflammasome, and MAPK cascades	[20,21]	Strong
Pro-Inflammatory Cytokine Signaling	Elevated release of TNF-α, IL-1β, IL-6, nitric oxide (NO), and reactive nitrogen species from activated glial cells	Synaptic remodeling, excitotoxicity, neuronal injury, and amplification of oxidative stress and neurodegeneration	AD, PD, ALS, and related proteinopathies	Phytochemicals may inhibit cytokine-mediated neurotoxicity through anti-inflammatory and immunomodulatory mechanisms	[22,23]	Moderate
Protein Misfolding and Aggregation	Amyloid-β oligomerization and plaque deposition	Disrupted synaptic signaling, impaired proteostasis, neurotoxicity, and neuronal network dysfunction	AD, mixed dementias	Natural compounds may inhibit amyloid aggregation, promote proteostasis, and enhance autophagic clearance	[24]	Strong
Tau Pathology	Hyperphosphorylation and aggregation of tau protein form neurofibrillary tangles	Cytoskeletal instability, impaired axonal transport, and synaptic dysfunction	AD, PSP, CBD, Pick’s disease	Phytochemicals may regulate kinase/phosphatase balance, reduce oxidative stress, and inhibit tau aggregation	[24]	Strong
Alpha-Synuclein Aggregation	Misfolding and aggregation of α-synuclein into Lewy bodies	Synaptic dysfunction, mitochondrial damage, and propagation of toxic protein species	PD, DLB, synucleinopathies	Phytochemicals may reduce α-synuclein aggregation and oxidative stress-mediated neurotoxicity	[25]	Moderate
Mitochondrial Dysfunction	Reduced ATP production, impaired oxidative phosphorylation, mitochondrial ROS generation, defective fusion–fission dynamics, impaired mitophagy	Energy deficit, calcium dysregulation, oxidative stress amplification, neuronal vulnerability	AD, PD, HD, ALS	Natural compounds may support mitochondrial biogenesis, restore redox balance, and enhance mitophagy	[26,27]	Strong
Synaptic Dysfunction and Loss	Disruption of synaptic signaling due to protein aggregates, inflammation, and metabolic stress	Impaired neurotransmission, reduced synaptic plasticity, and cognitive decline	AD, PD dementia, DLB	Phytochemicals may preserve synaptic integrity by reducing oxidative damage and inflammatory signaling	[28,29]	Strong
Neuronal Death	Activation of apoptotic, necroptotic, excitotoxic, and ER-stress-mediated cell death pathways	Progressive loss of neuronal populations and neural circuits	AD, PD, HD, ALS	Multi-target phytochemicals may interrupt upstream pathogenic cascades before irreversible neuronal degeneration	[19,30]	Moderate

Table Note: AD, Alzheimer’s disease; PD, Parkinson’s disease; HD, Huntington’s disease; ALS, amyotrophic lateral sclerosis; DLB, dementia with Lewy bodies; FTD, frontotemporal dementia; PSP, progressive supranuclear palsy; CBD, corticobasal degeneration; ROS, reactive oxygen species; RNS, reactive nitrogen species; NO, nitric oxide; TNF-α, tumour necrosis factor-α; IL-1β, interleukin-1β; IL-6, interleukin-6; NF-κB, nuclear factor-κB; NLRP3, NLR family pyrin domain containing 3; MAPK, mitogen-activated protein kinase; Nrf2, nuclear factor erythroid 2-related factor 2; Aβ, amyloid-β.

**Table 3:** Translational characteristics of representative neuroprotective phytochemicals: blood–brain barrier penetration, oral bioavailability, clinical development status in Alzheimer’s disease, and indicative technology readiness level (TRL).

Phytochemical/Class	Verified AD Translational Status	Verified BBB/CNS Evidence	Oral Bioavailability/PK Limitation	Original Data Verified	Evidence Strength/TRL	Citations
Galantamine/alkaloid AChE inhibitor	Approved symptomatic AD drug	Clinically CNS-active in humans; FDA label states efficacy supported by randomized placebo-controlled AD trials	Oral therapy; cholinergic adverse effects may limit dose	6-month RCT plus 6-month extension; improved cognition/global function in AD	High/TRL 9	[66]
Huperzine A/Lycopodium alkaloid AChE inhibitor	Phase II AD trial; not approved as standard AD drug	CNS-active AChE inhibitor; trial evidence in mild–moderate AD	Oral compound, but clinical efficacy uncertain	Phase II trial in mild–moderate AD: primary analysis showed no demonstrable cognitive benefit at 200 μg BID	Moderate but mixed/TRL 6	[67]
Resveratrol/stilbene polyphenol	Phase II AD biomarker trial completed	Human CSF evidence: resveratrol/metabolites penetrated BBB	Low free resveratrol exposure due to rapid metabolism	52-week randomized, placebo-controlled Phase II trial in mild–moderate AD; safe/tolerated; altered biomarker trajectories	Clinical biomarker/TRL 6	[42]
Curcumin/diarylheptanoid polyphenol	Small AD clinical trial; no established AD efficacy	Original AD trial did not prove robust brain-target engagement	Very low systemic bioavailability; extensive metabolism	36 mild–moderate AD participants; placebo, 2 g/day, or 4 g/day curcumin; 24-week randomized phase plus extension	Early clinical, inconsistent/TRL 5	[43]
EGCG/green-tea catechin	Mainly preclinical AD evidence	CNS/BBB evidence mainly animal/formulation-dependent; original paper used AD transgenic mice	Low stability and oral bioavailability; extensive metabolism	EGCG modulated APP cleavage and reduced cerebral amyloidosis in AD transgenic mice	Preclinical/TRL 3–4	[68]
Quercetin/flavonol	Preclinical AD evidence; no robust isolated-quercetin AD RCT	CNS effect shown in AD mice; human BBB evidence not established in cited paper	Poor solubility; extensive conjugation; low oral bioavailability	Chronic oral quercetin reduced β-amyloidosis/tauopathy and improved cognitive/emotional outcomes in aged 3xTg-AD mice	Preclinical/TRL 3–4	[48]
Kaempferol/flavonol	Preclinical dementia/AD-like evidence	CNS effect inferred from rodent hippocampal outcomes; direct human BBB data not established	Low solubility; metabolism limits exposure	STZ/ovariectomized rat model; kaempferol improved memory impairment and oxidative/neuroinflammatory markers	Preclinical/TRL 3	[69]
Berberine/isoquinoline alkaloid	Preclinical AD evidence	CNS activity shown in animal AD model; direct human AD BBB evidence not established	Very low oral bioavailability; P-gp efflux and first-pass metabolism are major limitations	Rabbit AD model; berberine reduced β-secretase/BACE-related activity and improved AD-like pathology indicators	Preclinical/TRL 3	[70]
Ginsenoside Rg1/ginseng saponin	Preclinical AD evidence	CNS benefit shown in AD models; human AD BBB data not established	Low oral absorption; gut microbial metabolism important	Aβ-induced/AD model evidence; Rg1 improved oxidative stress, apoptosis, and neuroinflammation via Wnt/GSK-3β/β-catenin pathway	Preclinical/TRL 3	[53]
Sulforaphane/isothiocyanate	Registered AD clinical trial; published representative evidence remains mainly preclinical	CNS effects shown in AD mice; clinical AD BBB data not yet established	Bioavailability depends on glucoraphanin conversion, myrosinase, gut microbiome	3xTg-AD mice; oral sulforaphane reduced Aβ/tau and improved memory; CHIP/HSP70 mechanism	Preclinical + trial registered/TRL 4–5	[71]
Luteolin/flavone	Preclinical AD evidence	CNS effect shown in 3xTg-AD mice; direct human BBB data not established	Low solubility and rapid metabolism; formulation-dependent	3xTg-AD mice and primary neurons; luteolin improved cognition, reduced Aβ generation, and repaired mitochondrial damage via PPARγ-dependent mechanism	Preclinical/TRL 3–4	[72]
Apigenin/flavone	Preclinical AD evidence	CNS effect shown in Aβ25–35 mouse model; direct human AD BBB data not established	Poor water solubility; rapid metabolism	Mouse Aβ25–35 toxicity model; apigenin protected neurovascular coupling and reduced Aβ-induced toxicity	Preclinical/TRL 3	[73]
Naringenin/citrus flavanone	Preclinical AD-like evidence	CNS effect shown in icv-STZ rat model; human BBB data not established	Better absorption than many flavonoids but extensive conjugation	icv-STZ rat model; naringenin reduced AD-type neurodegeneration and cognitive impairment	Preclinical/TRL 3	[74]
Fisetin/flavonol	Preclinical AD evidence	CNS effect shown in AD transgenic mice; human AD BBB evidence not established	Poor solubility; low/variable oral bioavailability	AD transgenic mice; fisetin maintained cognitive function and modulated p25/inflammatory pathways	Preclinical/TRL 3–4	[75]
Baicalein/flavone	In vitro/computational AD-target evidence; limited translational AD evidence	Article notes baicalein fractionally crossed BBB in prior evidence; original study mainly BACE1/AChE assays and in silico work	Oral exposure limited by metabolism/glucuronidation	In vitro and computational evidence for BACE1 and AChE inhibition	Early preclinical/TRL 2–3	[53]
Genistein/soy isoflavone	Small clinical trial in prodromal AD	Human clinical CNS outcome evidence; direct BBB quantification not central endpoint	Oral supplementation possible; conjugation affects free genistein levels	Double-blind placebo-controlled bicentric trial; 120 mg/day for 12 months in 24 prodromal AD patients; some cognitive-test improvements and amyloid-PET signal stabilization in anterior cingulate	Early clinical/TRL 5–6	[76]
Ferulic acid/phenolic acid	Preclinical AD evidence	CNS effect shown in APP/PS1 mice; direct human AD BBB data not established	More favorable small phenolic profile than many polyphenols but still rapidly metabolized	APP/PS1 mice; ferulic acid ameliorated AD-like pathology and cognitive decline by preventing capillary hypofunction	Preclinical/TRL 3–4	[77]
Astaxanthin/DHA-acylated astaxanthin/carotenoid	Preclinical AD evidence	CNS effects shown in APP/PS1 mice; BBB penetration not directly quantified in cited original AD paper	Lipophilic; absorption improves with dietary fat; formulation matters	APP/PS1 double-transgenic mice; astaxanthin-DHA showed stronger effects than astaxanthin on oxidative stress, tau hyperphosphorylation, neuroinflammation, and cognition	Preclinical/TRL 3–4	[78]
Lutein + zeaxanthin + meso-zeaxanthin/macular carotenoids	AD randomized supplementation trial	Retina/brain-related carotenoid evidence; clinical trial focused on macular pigment, vision, cognition	Fat-dependent absorption; interindividual variability	31 AD patients and 31 controls; active supplement increased serum carotenoids and macular pigment; visual function improved; cognitive outcomes did not significantly change over 6 months	Early clinical supportive/TRL 5	[79]
Saffron/crocin–crocetin-rich extract/carotenoid-derived phytochemicals	Phase II-style clinical evidence in mild–moderate AD	Clinical CNS outcome evidence for saffron extract; isolated crocin/crocetin BBB evidence varies and is not equivalent to whole extract	Crocin has poor direct absorption and is converted to crocetin; extract standardization matters	54 adults with mild–moderate AD; saffron 30 mg/day for 22 weeks showed effects similar to donepezil 10 mg/day; vomiting more frequent with donepezil	Clinical preliminary/TRL 6	[80]
Oleocanthal/olive-oil phenolic	Preclinical AD evidence	Strong BBB-model relevance: enhanced Aβ clearance across human BBB model and from TgSwDI mouse brain	Variable exposure; olive phenolics extensively metabolized	TgSwDI mice + in vitro human BBB model; oleocanthal enhanced Aβ clearance and reduced brain Aβ load	Preclinical/TRL 4	[81]
Withanoside IV/sominone/withanolide-related compounds	Preclinical AD evidence	CNS effect shown after oral withanoside IV in Aβ25–35 mice; sominone identified as active metabolite	Metabolic conversion to sominone appears important	Oral withanoside IV 10 μmol/kg/day improved memory deficits in Aβ25–35-injected mice and protected axons/dendrites/synapses; sominone promoted neurite/synapse reconstruction in vitro	Preclinical/TRL 3–4	[82]
Bacoside A/Bacopa monnieri saponin mixture	Mainly in vitro anti-amyloid evidence; Bacopa extracts have broader cognition literature, but isolated bacoside A is not an AD therapy	Direct BBB/human AD CNS data not established for isolated bacoside A	Complex mixture; extract standardization and bioavailability are major issues	Aβ42 cell/biophysical model; bacoside A inhibited β-amyloid cytotoxicity, fibrillation, and membrane interactions	Early preclinical/TRL 2–3	[83]
Urolithin A/ellagitannin gut-microbial metabolite	Recent strong preclinical AD evidence; no established AD clinical efficacy yet	CNS pharmacodynamic effects shown across AD mouse models; human AD BBB data not established	Exposure depends on gut microbiome conversion from ellagitannins or direct supplementation	Three AD mouse models; urolithin A improved cognition and restored mitophagy/lysosomal function	Preclinical/TRL 4	[84]

**Table 4:** Comparative summary of phytochemicals with mitochondrial-protective activity: compound, principal mitochondrial target/mechanism, and prevailing level of experimental evidence.

Compound	Verified Principal Target/Mechanism	Evidence Type/Model	AD Relevance	Use in Manuscript	Citations
Curcumin micelles	Mitochondrial membrane potential, respiratory control ratio, brain mitochondrial function	In vivo Thy1-APPSL AD mouse model	Direct AD model	Strong direct mitochondrial–AD evidence	[120]
Resveratrol	SIRT1–PGC-1α activation; mitochondrial oxidative phosphorylation and aerobic capacity	In vivo mouse metabolic model	Not AD-specific	Use only as supportive mitochondrial-biogenesis evidence, not AD-specific proof	[121]
EGCG	Aβ-induced mitochondrial dysfunction, mitochondrial ROS, mitochondrial membrane potential	In vitro AD/Aβ mitochondrial model	Direct AD-related cellular evidence	Direct preclinical mitochondrial evidence	[122]
Quercetin	Mitochondrial function and cognition	In vivo AD mouse model	Direct AD model	Strong direct mitochondrial–AD evidence	[123]
Luteolin	Aβ-induced mitochondrial dysfunction, oxidative stress, neuronal apoptosis, PPARγ signaling	3xTg-AD mice and primary neurons	Direct AD model	Strong direct mitochondrial–AD evidence	[72]
Berberine	Axonal mitochondrial membrane potential, ATP preservation, synaptic protection	In vitro Aβ neuronal model	Direct AD-related cellular evidence	Direct preclinical mitochondrial evidence	[124]
Ginsenoside Rg1	Mitochondrial membrane potential, ATP, cytochrome-c oxidase, cytochrome-c release	Primary cortical neurons exposed to oligomeric Aβ1–42	Direct AD-related cellular evidence	Direct preclinical mitochondrial evidence	[57]
Sulforaphane	HSP70/CHIP-mediated proteostasis; Aβ/tau clearance	3xTg-AD mice and primary neurons	Direct AD model, but mitochondrial link is indirect	Use as AD proteostasis evidence, not primary mitochondrial evidence	[71]
Astaxanthin	SIRT1–PGC-1α pathway, oxidative stress, cognitive deficits	AD models, in vivo/in vitro	Direct AD-related evidence	Mitochondrial-pathway supportive evidence	[125]
Urolithin A	Mitophagy, lysosomal function, mitochondrial quality control	Multiple AD mouse models	Direct AD model	Strong direct mitophagy–AD evidence	[84]
Honokiol	Mitochondrial SIRT3, energy metabolism, mitochondrial ROS	PS1V97L AD transgenic mice and neuronal AβO model	Direct AD model	Strong direct mitochondrial–AD evidence	[126]
Salidroside	NRF2/SIRT3 pathway, mitochondrial and neurite protection	AD mouse/cellular models	Direct AD model	Strong direct mitochondrial–AD evidence	[127]
Crocin	Mitochondrial damage, oxidative stress, memory deficit	Aβ-induced hippocampal rat model	Direct AD-like model	Direct mitochondrial–AD-like evidence	[128]
Carnosic acid	Aβ toxicity, cholinergic dysfunction, mitochondrial defects	C. elegans AD model	AD-like organism model	Direct preclinical mitochondrial evidence	[129]
Oleuropein aglycone	Autophagy-related clearance; mitochondrial protection is indirect	AD-related preclinical model	AD-related, but mitochondrial claim indirect	Use as autophagy/clearance evidence, not direct mitochondrial evidence	[130]
Hydroxytyrosol	Mitochondrial energetic dysfunction	7PA2 cellular AD model	Direct AD cellular model	Direct mitochondrial cellular evidence	[131]
Icariin	Mitochondrial transport, motility, mitochondrial index, length and size	Primary hippocampal neurons from 3xTg-AD mice	Direct AD cellular model	Direct mitochondrial–AD cellular evidence	[132]
Genistein	Mitochondrial apoptotic pathway: caspase-3, Bax, cytochrome-c	Rat AD model	Direct AD-like model	Direct mitochondrial-apoptosis evidence	[125]
Paeoniflorin	Mitochondrial membrane potential, cytochrome-c release, caspase-3/9	Aβ25–35-induced PC12 cell model	Direct AD cellular model	Direct mitochondrial cellular evidence	[133]
Puerarin	ROS, Bax/Bcl-2, oxidant-stress induced apoptosis	AD neuronal cybrid model	Direct AD cellular model	Direct mitochondrial/apoptosis evidence	[134]
Apigenin	Neurovascular coupling, oxidative stress protection; mitochondrial claim indirect	Aβ25–35 mouse model	Direct AD-like model, but not primarily mitochondrial	Use as AD neurovascular/oxidative evidence, not direct mitochondrial evidence	[73]
Naringenin	Oxidative stress and hippocampal neuronal injury	icv-STZ rat AD-type model	AD-like model	Mitochondrial claim should be indirect unless supported by additional study	[74]
Fisetin	PGC-1α, TFAM, mtDNA copy number, mitochondrial mass	SH-SY5Y neuronal cells	Not AD-specific	Use only as general neuronal mitochondrial-biogenesis evidence	[135]
Baicalein	Antioxidant/cholinergic protection; not primarily mitochondrial in cited study	AD-induced rat model	Direct AD-like model, but mitochondrial evidence indirect	Use as AD behavioral/oxidative evidence, not direct mitochondrial evidence	[136]
Ferulic acid	Mitochondrial bioenergetics, mitochondrial dynamics, Drp1, PGC-1α, mPTP	STZ-induced sporadic dementia/AD-type rat model	Direct AD-like model	Strong direct mitochondrial–AD-like evidence	[137]
Kaempferol	ER–mitochondria coupling and mitochondrial dysfunction	C9ORF72-ALS motor-neuron model	Not AD-specific	Do not present as AD evidence; include only if section covers broader neurodegeneration	[138]
Withanoside IV/sominone	Neurite/synapse reconstruction after Aβ injury; mitochondrial claim indirect	Aβ25–35 mouse model and cultured cortical neurons	Direct AD-like model	Use as neuronal reconstruction evidence, not direct mitochondrial evidence	[82]
Bacoside A	Aβ cytotoxicity, fibrillation, membrane interactions; mitochondrial claim indirect	In vitro Aβ42 biophysical/cellular model	AD-related in vitro evidence	Use as anti-amyloid evidence, not direct mitochondrial evidence	[83]

**Table 5:** Level-of-evidence matrix for representative phytochemicals in Alzheimer’s disease, differentiating in vitro, animal-model, early-phase/pilot clinical, and adequately powered randomised-controlled-trial (RCT) evidence. * Galantamine is an approved plant-derived alkaloid included for reference.

Phytochemical	In Vitro/Mechanistic Evidence	Animal-Model Evidence	Early Clinical/Phase I–II/Pilot Evidence	Robust Clinical Evidence	Highest Verified Evidence Level	Citations
Galantamine *	Yes; AChE inhibition	Yes	Yes	Yes; approved symptomatic AD therapy	Robust clinical	[66]
Huperzine A	Yes; AChE inhibition	Yes	Yes; Phase II AD trial, mixed/negative primary outcome	No	Early clinical, inconclusive	[67]
Curcumin	Yes; anti-Aβ, antioxidant, anti-inflammatory	Yes	Yes; small AD RCT	No	Early clinical, limited	[43]
Resveratrol	Yes; SIRT1/anti-inflammatory mechanisms	Yes	Yes; Phase II safety/biomarker AD trial	No	Early clinical, biomarker-focused	[42]
EGCG	Yes; APP processing/Aβ mechanisms	Yes	Limited/non-definitive	No	Strong preclinical	[68]
Quercetin	Yes; mechanistic preclinical support	Yes	No AD-specific clinical trial	No	Strong preclinical	[48]
Berberine	Yes; APP/BACE-related mechanisms	Yes	No AD-specific clinical trial	No	Preclinical	[70]
Luteolin	Yes; Aβ/oxidative stress/mitochondrial mechanisms	Yes	No	No	Strong preclinical	[72]
Ginsenoside Rg1	Yes; anti-apoptotic/anti-inflammatory mechanisms	Yes	Limited/non-definitive	No	Preclinical	[132]
Sulforaphane	Yes; proteostasis/CHIP/HSP pathway	Yes	Registered/early interest; no definitive AD efficacy	No	Preclinical/translational candidate	[71]
Genistein	Yes; antioxidant/estrogenic mechanisms	Yes	Yes; prodromal AD clinical trial	No	Early clinical, preliminary	[76]
Saffron/crocin–crocetin-rich extract	Yes; anti-Aβ/antioxidant mechanisms	Yes	Yes; small AD RCT	No confirmatory robust RCT	Early clinical, promising but preliminary	[80]
Macular carotenoids: lutein, zeaxanthin, meso-zeaxanthin	Yes; antioxidant/neuroretinal support	Yes	Yes; small AD supplementation RCT	No	Early clinical supportive	[79]
Astaxanthin/AST-DHA	Yes; antioxidant/anti-inflammatory mechanisms	Yes	No AD-specific clinical trial	No	Preclinical	[78]
Oleocanthal	Yes; BBB/Aβ transport model	Yes	No AD-specific clinical trial	No	Preclinical, BBB/Aβ-clearance focused	[81]
Urolithin A	Yes; mitophagy/lysosomal mechanisms	Yes	No AD-specific clinical trial	No	Strong recent preclinical	[84]
Apigenin	Yes; antioxidant/neurovascular mechanisms	Yes	No AD-specific clinical trial	No	Preclinical	[73]
Naringenin	Yes; antioxidant/anti-inflammatory mechanisms	Yes	No AD-specific clinical trial	No	Preclinical	[74]
Fisetin	Yes; p25/inflammatory pathways	Yes	No AD-specific clinical trial	No	Preclinical	[75]
Baicalein	Yes; BACE1/AChE inhibition and docking	Limited/preclinical	No AD-specific clinical trial	No	In vitro/computational + preclinical	[132]
Ferulic acid	Yes; anti-Aβ/oxidative mechanisms	Yes	No AD-specific clinical trial	No	Preclinical	[77]
Kaempferol	Yes; antioxidant/neuroinflammatory mechanisms	Yes; AD-like/sporadic dementia model	No AD-specific clinical trial	No	Preclinical/AD-like	[69]
Withanoside IV/sominone	Yes; neurite/synapse reconstruction	Yes; Aβ25–35 model	No AD-specific clinical trial	No	Preclinical	[82]
Bacoside A	Yes; Aβ42 cytotoxicity/fibrillation/membrane interaction	Limited/extract-based	No AD-specific clinical trial	No	Mainly in vitro/early preclinical	[83]

**Table 6:** Therapeutic Potential of Major Phytochemicals in Alzheimer’s Disease.

Phytochemical	Mechanism of Action	Experimental Model	Key Findings	Citation
Curcumin	Antioxidant; reduces ROS and lipid peroxidation	Transgenic AD mice	Reduced oxidative damage and amyloid pathology	[175]
	Inhibits amyloid-β aggregation and fibril formation	In vitro and mouse model	Bound plaques and reduced amyloid accumulation	[176]
	Anti-inflammatory via PPAR-γ activation	Rat AD model	Reduced neuroinflammation and neuronal damage	[177]
	Modulation of multiple signaling pathways	Review study	Described mechanisms targeting amyloid and tau pathology	[148]
	Brain-targeted delivery and neuroprotective signaling	Review	Curcumin modulates PI3K/Akt and Wnt pathways	[178]
	Improves cognition and reduces amyloid and tau	Human study in non-demented older adults	Memory improvements in older adults	[179]
Resveratrol	Activates SIRT1 signaling and mitochondrial protection	Cellular models	Reduced oxidative stress and neuronal death	[180]
	Anti-inflammatory and anti-amyloid effects	Clinical trial	Evaluated safety and biomarkers in AD patients	[42]
	Combination therapy improves cognitive markers	Clinical pilot study	Improved AD biomarkers	[181]
	Antioxidant and neuroprotective effects	Systematic review	Summarized therapeutic potential	[182]
	Modulates amyloid metabolism and inflammation	Review	Supports the neuroprotective role in AD	[183]
Quercetin	Antioxidant; scavenges ROS	Review	Protects neurons against oxidative stress	[49]
	Reduces neurodegeneration and inflammation	Animal AD models	Improved cognition and neuronal survival	[184]
	Pharmacokinetics and nano-delivery systems	Review	Improved brain bioavailability	[185]
	Modulates molecular signaling pathways	Review	Regulates inflammatory and oxidative pathways	[186]
	Reduces oxidative stress and mitochondrial damage	Cellular AD models	Improved neuronal survival	[187]
EGCG	Promotes non-amyloidogenic APP processing	Transgenic AD mice	Reduced cerebral amyloidosis	[68]
	Remodels amyloid fibrils and reduces toxicity	Molecular study	Reduced the toxicity of amyloid fibrils	[147]
	Anti-inflammatory and antioxidant effects	Review	EGCG modulates multiple AD pathways	[188]
	Modulates amyloid aggregation	Review	Demonstrated protective effects in neurodegeneration	[173]
	Translational perspective for AD therapy	Review	Evaluates potential clinical use	[189]

**Table 7:** Chemical identity of representative phytochemicals discussed in this review, including chemical class, representative source, molecular formula, and PubChem CID.

Phytochemical	Chemical class	Representative source	Molecular formula	PubChem CID
Curcumin	Diarylheptanoid polyphenol	Curcuma longa/turmeric	C21H20O6	969516
Resveratrol	Stilbene polyphenol	Grapes, berries, peanuts	C14H12O3	445154
EGCG	Catechin polyphenol	Green tea/Camellia sinensis	C22H18O11	65064
Quercetin	Flavonol	Onions, apples, berries	C15H10O7	5280343
Luteolin	Flavone	Celery, parsley, chamomile	C15H10O6	5280445
Kaempferol	Flavonol	Tea, kale, broccoli	C15H10O6	5280863
Apigenin	Flavone	Parsley, celery, chamomile	C15H10O5	5280443
Naringenin	Flavanone	Citrus fruits, grapefruit	C15H12O5	439246
Fisetin	Flavonol	Strawberries, apples, onions	C15H10O6	5281614
Baicalein	Flavone	*Scutellaria baicalensis*	C15H10O5	5281605
Genistein	Isoflavone	Soybean and soy products	C15H10O5	5280961
Berberine	Protoberberine alkaloid	*Berberis* spp., *Coptis* spp.	C20H18NO4+	2353
Galantamine	Amaryllidaceae alkaloid	*Galanthus* and *Narcissus* spp.	C17H21NO3	9651
Huperzine A	Lycopodium alkaloid	Huperzia serrata	C15H18N2O	854026
Ginsenoside Rg1	Triterpenoid saponin	Panax ginseng	C42H72O14	441923
Sulforaphane	Isothiocyanate	Broccoli and other cruciferous vegetables	C6H11NOS2	5350
Astaxanthin	Carotenoid/xanthophyll	Microalgae, salmon, shrimp	C40H52O4	5281224
Lutein	Carotenoid/xanthophyll	Leafy green vegetables, egg yolk	C40H56O2	5281243
Zeaxanthin	Carotenoid/xanthophyll	Maize, goji berry, egg yolk	C40H56O2	5280899
Crocin	Apocarotenoid glycoside	Saffron/Crocus sativus	C44H64O24	5281233
Crocetin	Apocarotenoid dicarboxylic acid	Saffron/Crocus sativus	C20H24O4	5281232
Ferulic acid	Phenolic acid	Rice bran, wheat bran, cereals	C10H10O4	445858
Hydroxytyrosol	Phenylethanoid/catechol phenol	Olive oil, olive fruit	C8H10O3	82755
Oleocanthal	Secoiridoid-derived phenolic compound	Extra-virgin olive oil	C17H20O5	11652416
Urolithin A	Ellagitannin-derived gut microbial metabolite	Produced from ellagitannins in pomegranate, berries, walnuts	C13H8O4	5488186
Honokiol	Biphenolic neolignan	*Magnolia* spp.	C18H18O2	72303
Salidroside	Phenylethanoid glycoside	Rhodiola rosea	C14H20O7	159278
Carnosic acid	Phenolic diterpene	Rosemary, sage	C20H28O4	65126
Icariin	Prenylated flavonol glycoside	*Epimedium* spp.	C33H40O15	5318997
Paeoniflorin	Monoterpene glycoside	Paeonia lactiflora	C23H28O11	442534
Puerarin	Isoflavone C-glycoside	Pueraria lobata/kudzu root	C21H20O9	5281807
Withanoside IV	Withanolide glycoside	Withania somnifera	C40H62O15	71312551
Bacoside A/bacoside-rich fraction	Triterpenoid saponin	Bacopa monnieri	C41H68O13	92043183

**Table 8:** Clinical and AD-relevant preclinical evidence for representative phytochemicals in Alzheimer’s disease, including study design, population or model, main outcome, and translational status.

Compound/Intervention	Study Design	Population/Model	Short Outcome	Translational Status	Citations
Galantamine * (* means positive control)	6-month randomized placebo-controlled trial + extension	AD patients	Improved cognition and global function	Approved symptomatic AD therapy	[66]
Huperzine A	Phase II randomized placebo-controlled trial	Mild–moderate AD patients	No clear benefit at 200 μg twice daily	Clinical evidence inconclusive	[67]
Curcumin C3 Complex^®^	24-week randomized double-blind placebo-controlled trial	Mild–moderate AD patients	Safe, but no clear clinical or biomarker benefit	Negative/limited AD trial	[43]
Bioavailable curcumin/Theracurmin^®^	18-month double-blind placebo-controlled trial	Non-demented older adults, not AD patients	Improved memory and attention; reduced amyloid/tau PET signal	AD-relevant, but not AD-patient trial	[179]
Resveratrol	52-week phase II randomized double-blind placebo-controlled trial	Mild–moderate AD patients	Safe; entered CNS; changed some biomarkers; no proven clinical efficacy	Completed biomarker/safety trial	[42]
Resveratrol + glucose/malate	Randomized double-blind pilot trial	Mild–moderate AD patients	Exploratory study; not confirmatory	Pilot clinical evidence	[181]
Trans-resveratrol	Randomized double-blind clinical trial	Mild–moderate AD patients	Reported possible neuroprotective benefit; needs replication	Exploratory clinical evidence	[193]
Saffron extract	16-week randomized placebo-controlled trial	Mild–moderate AD patients	Improved cognitive scores versus placebo	Small positive clinical trial	[194]
Saffron extract vs. donepezil	22-week randomized double-blind trial	Mild–moderate AD patients	Similar effect to donepezil; donepezil caused more vomiting	Promising comparative trial	[194]
Saffron extract vs. memantine	1-year double-blind randomized trial	Moderate–severe AD patients	Similar effect to memantine	Promising, but needs larger trials	[195]
Saffron add-on to donepezil	12-week randomized placebo-controlled trial	Donepezil-treated mild–moderate AD patients	Improved inflammation/oxidative markers; no clear cognitive benefit	Biomarker benefit only	[196]
Genistein	Double-blind placebo-controlled clinical trial	Prodromal AD patients	Possible cognitive/PET benefit; larger trials needed	Early clinical evidence	[76]
Macular carotenoids: lutein, zeaxanthin, meso-zeaxanthin	6-month randomized clinical trial	AD patients and controls	Improved visual function; no clear cognitive benefit	Supportive early clinical evidence	[79]
Melissa officinalis extract	4-month randomized placebo-controlled trial	Mild–moderate AD patients	Improved cognition/global scores; reduced agitation	Small positive trial	[197]
Melissa officinalis extract rich in rosmarinic acid	24-week randomized placebo-controlled trial	Mild dementia due to AD	Safe; possible neuropsychiatric benefit	Early supportive evidence	[198]
Salvia officinalis extract	Randomized placebo-controlled trial	Mild–moderate AD patients	Improved AD symptoms in a small trial	Small positive trial	[197]
Ginkgo biloba extract EGb 761	52-week randomized placebo-controlled trial	AD or vascular dementia patients	Modest cognitive/social benefit	Clinical evidence for extract, not isolated compound	[199]
Ginkgo biloba extract vs. donepezil/placebo	Randomized placebo-controlled double-blind trial	Alzheimer-type dementia patients	Benefit appeared comparable to donepezil	Comparative clinical evidence	[200]
EGCG	Preclinical in vivo AD transgenic mouse study	AD transgenic mice	Reduced brain amyloid burden	Preclinical AD evidence; clinical efficacy not proven	[68]
Quercetin	Preclinical in vivo AD model	Aged 3xTg-AD mice	Reduced Aβ, tau, gliosis; improved memory	Strong preclinical AD evidence	[48]
Berberine	Preclinical in vivo AD-like model	Rabbit AD model	Reduced β-secretase-related AD changes	Preclinical AD evidence	[70]
Luteolin	Preclinical in vivo and in vitro AD study	3xTg-AD mice and neurons	Improved memory; reduced Aβ, oxidative stress, mitochondrial damage	Strong preclinical AD evidence	[72]
Sulforaphane	Preclinical in vivo and in vitro AD study	3xTg-AD mice and primary neurons	Reduced Aβ/tau and improved memory-related outcomes	Preclinical/translational candidate	[71]
Astaxanthin/DHA-acylated astaxanthin	Preclinical in vivo AD model	APP/PS1 mice	Improved cognition; reduced oxidative stress, tau phosphorylation, and inflammation	Preclinical AD evidence	[78]
Urolithin A	Preclinical in vivo AD models	Multiple AD mouse models	Improved memory; restored mitophagy and lysosomal function	Strong recent preclinical AD evidence	[84]
Apigenin	Preclinical in vivo AD-like model	Aβ25–35 mouse model	Protected brain blood-flow signaling and reduced Aβ toxicity	Preclinical AD-like evidence	[73]
Naringenin	Preclinical in vivo AD-like model	icv-STZ rat model	Improved memory; reduced neuronal injury and oxidative stress	Preclinical AD-like evidence	[74]
Fisetin	Preclinical in vivo AD model	AD transgenic mice	Preserved cognition; reduced p25/inflammatory signaling	Preclinical AD evidence	[75]
Baicalein	In vitro/computational AD-target study	BACE1 and AChE assay/docking models	Inhibited BACE1 and AChE targets	Early preclinical/mechanistic evidence	[132]
Ferulic acid	Preclinical in vivo AD model	APP/PS1 mice	Improved cognition and AD-like pathology	Preclinical AD evidence	[77]
Kaempferol	Preclinical AD-like dementia model	STZ/ovariectomized rat model	Improved memory; reduced oxidative stress and inflammation	Preclinical AD-like evidence	[69]
Withanoside IV/sominone	Preclinical in vivo and in vitro AD-like study	Aβ25–35 mouse model and neurons	Improved memory; supported neurite/synapse recovery	Preclinical AD-like evidence	[82]
Bacoside A	In vitro Aβ42 study	Aβ42 cell/biophysical model	Reduced Aβ toxicity, fibril formation, and membrane damage	Early preclinical evidence	[83]
Crocin	Preclinical in vivo AD-like model	Aβ-induced hippocampal rat model	Protected mitochondria and improved memory deficit	Preclinical AD-like evidence	[128]
Honokiol	Preclinical in vivo AD model	PS1V97L AD mice	Improved cognition; activated mitochondrial SIRT3	Preclinical AD evidence	[126]
Salidroside	Preclinical in vivo AD model	5xFAD mice and Aβ models	Reduced AD pathology through NRF2/SIRT3 signaling	Preclinical AD evidence	[127]
Icariin	Preclinical cellular AD model	Hippocampal neurons from 3xTg-AD mice	Improved mitochondrial transport in neurons	Preclinical AD cellular evidence	[132]
Paeoniflorin	Preclinical in vitro AD model	Aβ25–35-treated PC12 cells	Protected cells from mitochondrial damage	Early preclinical evidence	[133]
Puerarin	Preclinical in vitro AD model	AD neuronal cybrid cells	Reduced oxidative-stress-induced cell death	Early preclinical evidence	[134]
Hydroxytyrosol	Preclinical cellular AD model	7PA2 AD cellular model	Improved mitochondrial energy function	Early preclinical AD cellular evidence	[131]

**Table 9:** Evidence from Experimental and Clinical Studies for Alzheimer’s Disease. (**a**) Animal Studies (Rodent Models of Neurodegeneration). (**b**) Clinical Trials (Existing Studies).

Study	Model/Population	Methods	Key Findings	Limitations	citation
Probing sporadic and familial AD using induced pluripotent stem cells	Human iPSC-derived neurons from sporadic and familial AD patients	iPSC reprogramming, neuronal differentiation, AD phenotype analysis	Demonstrated that both sporadic and familial AD can be modeled in human neurons; showed increased Aβ and stress-related phenotypes	In vitro model lacks a full brain microenvironment and aging context	[211]
Modeling AD with iPSCs reveals stress phenotypes associated with intracellular Aβ and differential drug responsiveness	Human iPSC-derived neural cells	Patient-derived iPSCs, intracellular Aβ analysis, drug testing	Showed intracellular Aβ-associated stress and variable response to candidate drugs	Limited sample diversity and in vitro maturation state	[212]
The familial AD APPV717I mutation alters APP processing and Tau expression in iPSC-derived neurons	Human iPSC-derived neurons with the APP mutation	Mutation-specific neuronal modeling, APP processing assays, tau expression analysis	Identified altered APP processing and abnormal tau expression linked to familial AD mutation	Single mutation model; limited generalizability to sporadic AD	[213]
A three-dimensional human neural cell culture model of AD	3D human neural culture	3D culture system, amyloid and tau pathology assessment	Landmark study showing both extracellular amyloid aggregation and tau pathology in one human system	Artificial culture system, not equivalent to intact brain architecture	[214]
Self-organizing 3D human neural tissue derived from induced pluripotent stem cells recapitulates AD phenotypes	Human iPSC-derived 3D neural tissue	3D differentiation, tissue self-organization, AD phenotype analysis	Reproduced AD-like phenotypes in a human neural tissue model	Long culture times and variable reproducibility	[215]
APOE4 causes widespread molecular and cellular alterations associated with AD phenotypes in human iPSC-derived brain cell types	Human iPSC-derived neurons and glia	Isogenic APOE models, transcriptomic and phenotypic analysis	Showed APOE4 induces broad neuronal and glial abnormalities relevant to AD	A genetic model may not capture the full disease heterogeneity	[216]
Modeling amyloid beta and tau pathology in human cerebral organoids	Human cerebral organoids	Organoid generation, amyloid, and tau pathology detection	Demonstrated amyloid-β and tau pathology in cerebral organoids	Organoids remain developmentally immature and lack vasculature	[217]
A 3D human triculture system modeling neurodegeneration and neuroinflammation in AD	Human triculture of neurons, astrocytes, and microglia	3D triculture platform, inflammatory and degenerative phenotype assays	Reproduced neurodegeneration and neuroinflammation in a multicellular AD model	Still simplified compared with in vivo brain networks	[218]
Amyloid-β42/40 ratio drives tau pathology in 3D human neural cell culture models of AD	3D human neural cultures	Amyloid ratio manipulation, tau pathology assays	Found that the Aβ42/40 ratio strongly drives tau-related pathology	Focused on one mechanistic pathway in vitro	[219]
Altered ubiquitin signaling induces AD-like hallmarks in a three-dimensional human neural cell culture model	3D human neural culture	Proteostasis manipulation, amyloid, and tau assessment	Linked altered ubiquitin signaling with amyloid-like and tau-like pathology	Experimental perturbation may not fully reflect human disease progression	[220]
(**a**) Animal Studies (Rodent Models of Neurodegeneration)					
Correlative memory deficits, Aβ elevation, and amyloid plaques in transgenic mice	Tg2576 mouse model	Behavioral testing, plaque measurement, Aβ quantification	Classic evidence linking amyloid accumulation with memory deficits	Primarily amyloid-focused; incomplete tau pathology	[221]
Triple-transgenic model of AD with plaques and tangles: Intracellular Aβ and synaptic dysfunction	3xTg-AD mice	Triple transgenic design, histology, and synaptic function testing	Important model showing both amyloid and tau pathology	The mouse disease course differs from human AD progression	[222]
Intraneuronal β-amyloid aggregates, neurodegeneration, and neuron loss in transgenic mice with five familial AD mutations	5xFAD mice	Aggressive amyloid transgenic mouse model, pathology, and neuron loss assays	Rapid plaque development and neurodegeneration; widely used amyloid model	A very aggressive phenotype may overrepresent familial AD	[223]
Neurofibrillary tangles, amyotrophy and progressive motor disturbance in mice expressing mutant (P301L) tau protein	P301L tau transgenic mice	Tau transgenesis, histopathology, motor phenotype analysis	Landmark tauopathy model demonstrating neurofibrillary pathology	More representative of tauopathy than full AD	[224]
Transmission and spreading of tauopathy in the transgenic mouse brain	Tau transgenic mice	Tau seeding and propagation experiments	Provided evidence for the spreading of tau pathology in vivo	Seeded transmission may not fully mimic spontaneous disease	[225]
Propagation of tau pathology in a model of early AD	Mouse model of early tau pathology	Histological tracing of tau spread	Demonstrated trans-neuronal propagation of tau pathology	Does not fully explain the initiating triggers	[226]
Complement and microglia mediate early synapse loss in Alzheimer’s mouse models	AD mouse models	Synapse quantification, complement pathway analysis, and microglial assays	Showed that complement and microglia drive early synapse loss	Rodent immune responses may differ from those of humans	[227]
The neuritic plaque facilitates pathological conversion of tau in an AD mouse model	Amyloid mouse plus tau model	Amyloid-tau interaction studies, histopathology	Demonstrated that neuritic plaques promote pathological tau conversion	The mechanistic model does not capture full clinical heterogeneity	[228]
Selective removal of astrocytic APOE4 strongly protects against tau-mediated neurodegeneration and decreases synaptic phagocytosis by microglia	APOE4 tauopathy mouse model	Astrocyte-specific APOE4 removal, neurodegeneration assays	Showed astrocytic APOE4 is a major driver of tau-mediated degeneration	Genetic intervention may not be easily translatable clinically	[229]
Targeted BACE-1 inhibition in microglia enhances amyloid clearance and improves cognitive performance	5xFAD mouse with microglial targeting	Cell-specific BACE1 inhibition, cognition, and pathology assessment	Improved amyloid clearance and cognition through microglial targeting	Preclinical strategy is still far from human validation	[136]
(**b**) Clinical Trials (Existing Studies)					
A controlled trial of selegiline, alpha-tocopherol, or both as treatment for AD	Patients with AD	Randomized controlled trial	Early major trial examining the effects of disease progression of antioxidant and selegiline therapy	Older trial design; not disease-modifying by modern standards	[230]
A 24-week, double-blind, placebo-controlled trial of donepezil in patients with AD	Mild to moderate AD patients	Double-blind placebo-controlled trial	Established symptomatic cognitive benefit of donepezil	Symptomatic benefit only, not disease-modifying	[206]
Memantine treatment in patients with moderate to severe AD already receiving donepezil	Moderate to severe AD patients	Randomized controlled trial	Demonstrated symptomatic benefit of memantine in more advanced disease	Benefits were modest and symptomatic	[207]
Clinical effects of Aβ immunization (AN1792) in patients with AD in an interrupted trial	Patients with AD	Active immunization trial	First major amyloid immunization study; reduced plaque burden, but had safety concerns	Trial interrupted because of meningoencephalitis	[231]
A phase 3 trial of semagacestat for the treatment of AD	Mild to moderate AD patients	Phase 3 randomized trial	Failed to improve outcomes and raised safety concerns	Demonstrated risks of γ-secretase inhibition	[92]
Two phase 3 trials of bapineuzumab in mild-to-moderate AD	Mild to moderate AD patients	Phase 3 randomized trials	No meaningful clinical benefit despite the amyloid-targeting approach	Clinical efficacy did not match the biomarker rationale	[232]
Phase 3 trials of solanezumab for mild-to-moderate AD	Mild to moderate AD patients	Two phase 3 randomized trials	Failed to significantly improve primary clinical outcomes	Negative trial despite major therapeutic interest	[92]
The antibody aducanumab reduces Aβ plaques in AD	Early AD patients	Phase 1b clinical trial	Demonstrated dose-dependent amyloid reduction and renewed anti-amyloid momentum	Early-phase study, not definitive for clinical efficacy; accelerated FDA approval (2021) was contested, Phase 3 trials (EMERGE/ENGAGE) were discordant, and the antibody was withdrawn/discontinued in 2024	[233]
Donanemab in early AD	Early symptomatic AD patients	Phase 2 randomized trial	Showed slowing on integrated cognition/function endpoints	Safety issues, including ARIA, remained important	[234]
Lecanemab in early AD	Early AD patients	Phase 3 randomized trial	Demonstrated modest but statistically significant slowing of clinical decline	Benefit modest; ARIA and infusion-related adverse effects require monitoring	[235]

## Data Availability

No new data were created or analyzed in this study. Data sharing is not applicable to this article.
